# Supplementary material for: A fungal RNA-dependent RNA polymerase is a novel player in plant infection and cross-kingdom RNA interference
Source: PLoS Pathog. 2023 Dec 20;19(12):e1011885. doi: 10.1371/journal.ppat.1011885 (PMC10766185; doi:10.1371/journal.ppat.1011885)
Supplement: S1 Fig — Fungal RDR phylogenetic analysis (A) and amino acid sequence alignment of the RDR active site (B). The bar in (A) represents length of branch. Min and max refers to levels of conservation in (B). Amino acids sequences used in this analysis are given in S2 Table. (PDF) [file ppat.1011885.s001.pdf]

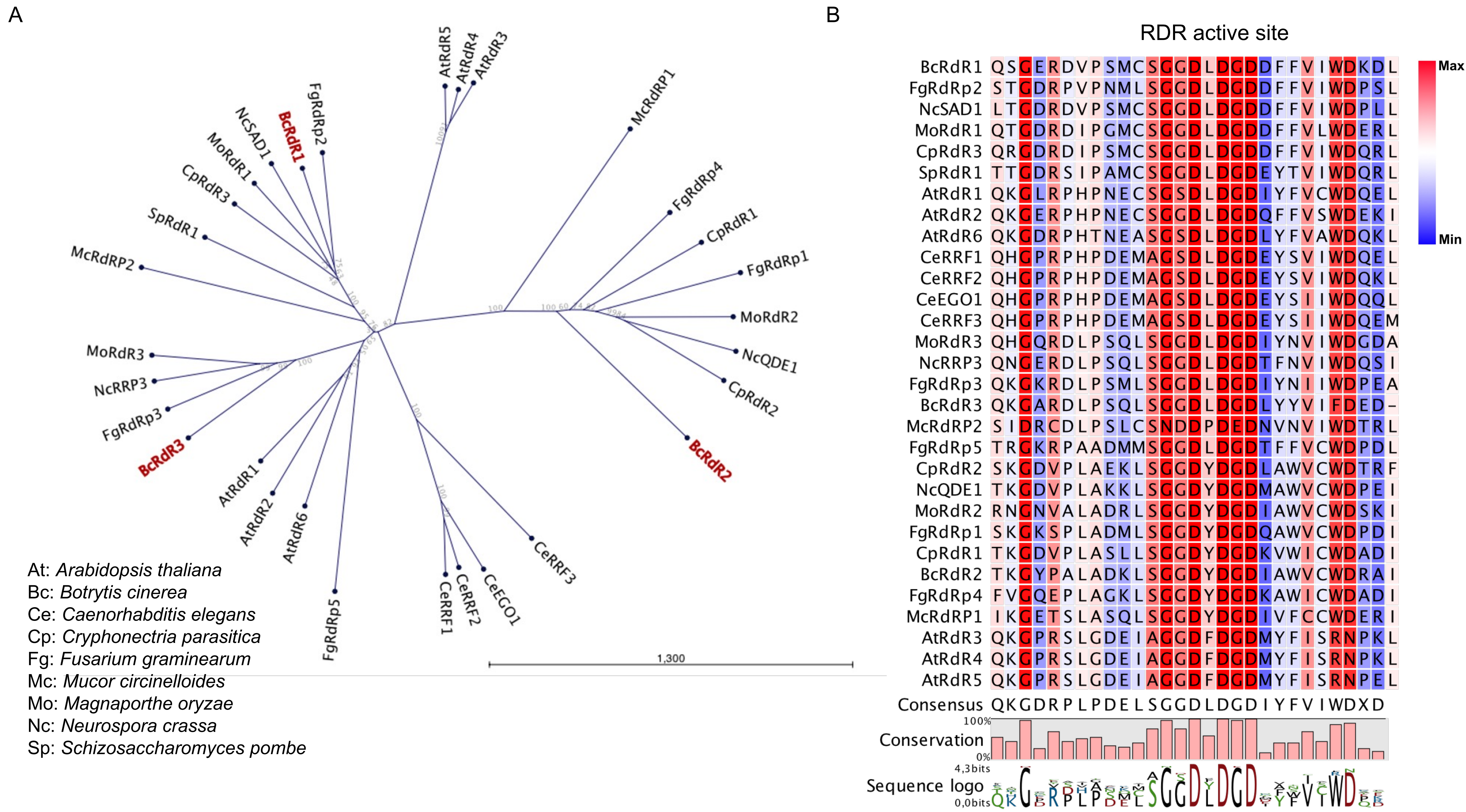
A
B
AtRdR5
AtRdR4
AtRdR3
McRdRP1
FgRdRp2
BcRdR1
NcSAD1
MoRdR1
CpRdR3
SpRdR1
McRdRP2
FgRdRp4
CpRdR1
FgRdRp1
MoRdR2
NcQDE1
CpRdR2
BcRdR2
MoRdR3
NcRRP3
FgRdRp3
BcRdR3
AtRdR1
AtRdR2
AtRdR6
FgRdRp5
CeRRF1
CeRRF2
CeEGO1
CeRRF3
1,300
At: *Arabidopsis thaliana*
Bc: *Botrytis cinerea*
Ce: *Caenorhabditis elegans*
Cp: *Cryphonectria parasitica*
Fg: *Fusarium graminearum*
Mc: *Mucor circinelloides*
Mo: *Magnaporthe oryzae*
Nc: *Neurospora crassa*
Sp: *Schizosaccharomyces pombe*
RDR active site
BcRdR1 QSGERDVPSMCSGGDLDGDDFFVIWDKDL
FgRdRp2 STGDRPVPNMLSGGDLDGDDFFVIWDPSL
NcSAD1 LTGDRDVPSMCSGGDLDGDDFFVIWDPLL
MoRdR1 QTGDRDIPGMCSGGDLDGDDFFVLWDERL
CpRdR3 QRGDRDIPSMCSGGDLDGDDFFVIWDQRL
SpRdR1 TTGDRSIPAMCSGGDLDGDEYTVIWDQRL
AtRdR1 QKGLRPHPNECSGSDLDGDIYFVCWDQEL
AtRdR2 QKGERPHPNECSGGDLDGDQFFVSWDEKI
AtRdR6 QKGDRPHTNEASGSDLDGDLYFVAWDQKL
CeRRF1 QHGPRPHPDEMAGSDLDGDEYSVIWDQEL
CeRRF2 QHGPRPHPDEMAGSDLDGDEYSVIWDQKL
CeEGO1 QHGPRPHPDEMAGSDLDGDEYSIIWDQQL
CeRRF3 QHGPRPHPDEMAGSDLDGDEYSIIWDQEM
MoRdR3 QHGQRDLPSQLSGGDLDGDIYNVIWDGDA
NcRRP3 QNGERDLPSQLSGGDLDGDTFNVIWDQSI
FgRdRp3 QKGKRDLPSMLSGGDLDGDIYNIIWDPEA
BcRdR3 QKGARDLPSQLSGGDLDGDLYYVIFDED-
McRdRP2 SIDRCDLPSLCSNDDPDEDNVNVIWDTRL
FgRdRp5 TRGKRPAADMMSGGDLDGDTFFVCWDPDL
CpRdR2 SKGDVPLAEKLSGGDYDGDLAWVCWDTRF
NcQDE1 TKGDVPLAKKLSGGDYDGDMAWVCWDPEI
MoRdR2 RNGNVALADRLSGGDYDGDIAWVCWDSKI
FgRdRp1 SKGKSPLADMLSGGDYDGDQAWVCWDPDI
CpRdR1 TKGDVPLASLLSGGDYDGDKVWICWDADI
BcRdR2 TKGYPALADKLSGGDYDGDIAWVCWDRAI
FgRdRp4 FVGQEPLAGKLSGGDYDGDKAWICWDADI
McRdRP1 IKGETSLASQLSGGDYDGDIVFCCWDERI
AtRdR3 QKGPRSLGDEIAGGDFDGDMYFISRNPKL
AtRdR4 QKGPRSLGDEIAGGDFDGDMYFISRNPKL
AtRdR5 QKGPRSLGDEIAGGDFDGDMYFISRNPEL
Consensus QKGDRPLPDELSGGDLDGDIYFVIWDXD
Max
Min
Conservation
100%
0%
Sequence logo
4,3bits
0,0bits
