# Supplementary figures and images for: A fungal RNA-dependent RNA polymerase is a novel player in plant infection and cross-kingdom RNA interference
Source: PLoS Pathog. 2023 Dec 20;19(12):e1011885. doi: 10.1371/journal.ppat.1011885 (PMC10766185; doi:10.1371/journal.ppat.1011885)

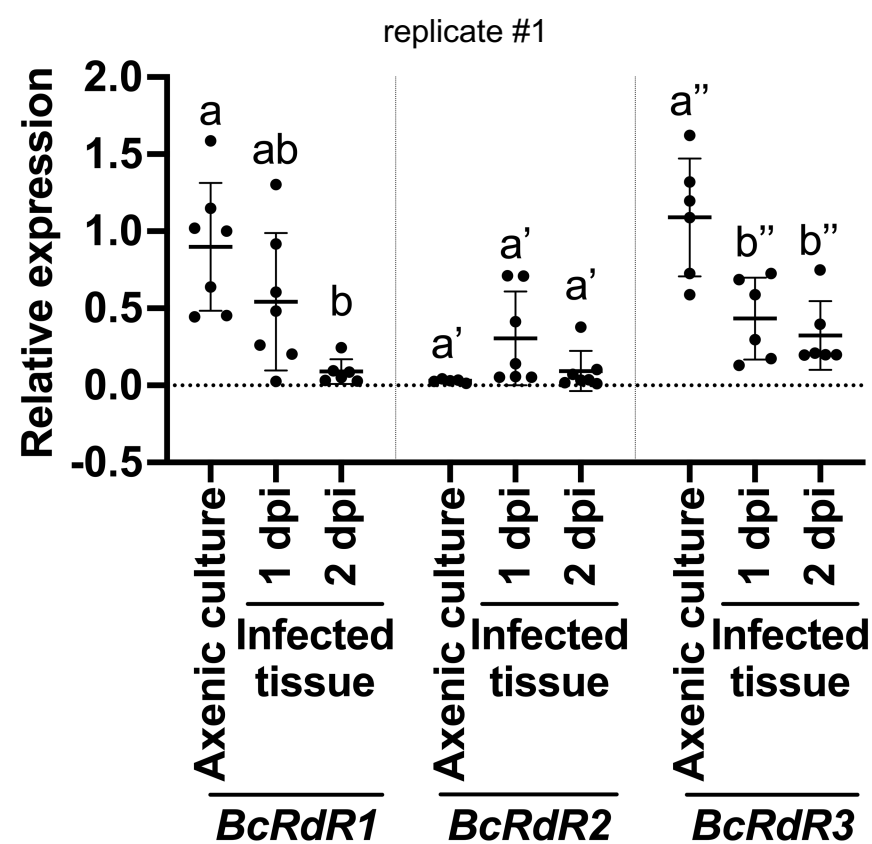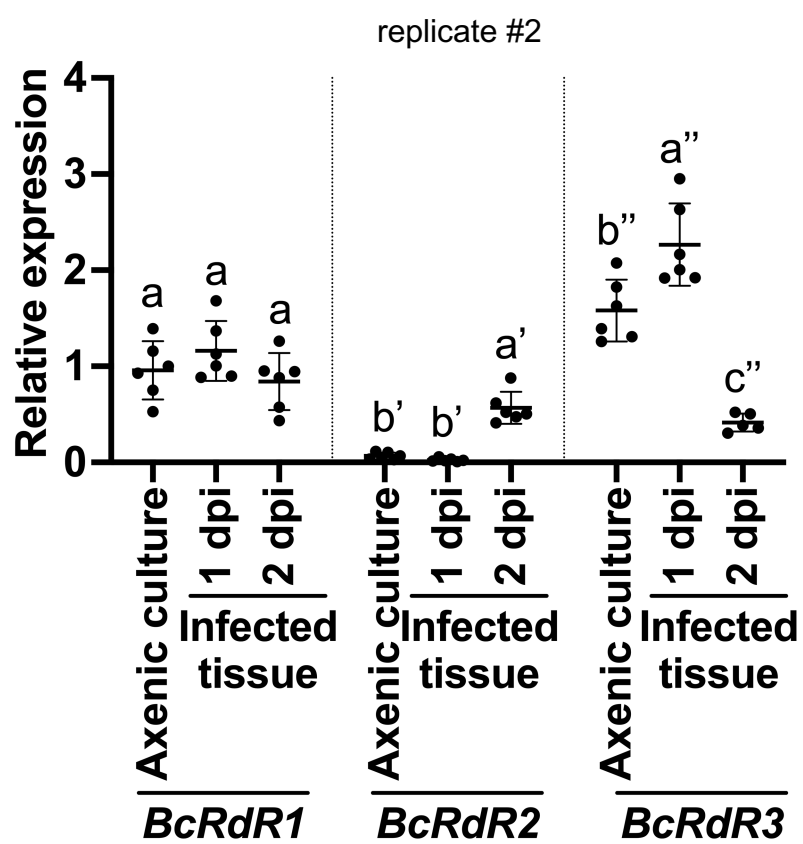

Supplement: S2 Fig — BcRDRs mRNA levels were measured in two independent replicates by qRT-PCR using the BcTubA as a reference gene. Lines in scatter plots represent the mean and the standard deviation. Statistical analysis was performed using ANOVA followed by a Tukey post-hoc test with p-value threshold p < 0.05. (PDF) [file ppat.1011885.s002.pdf]

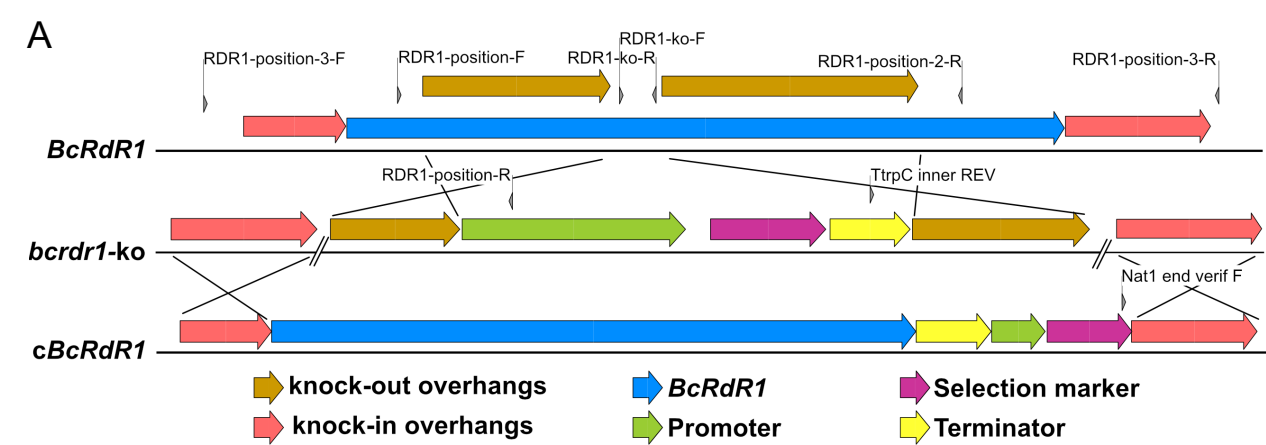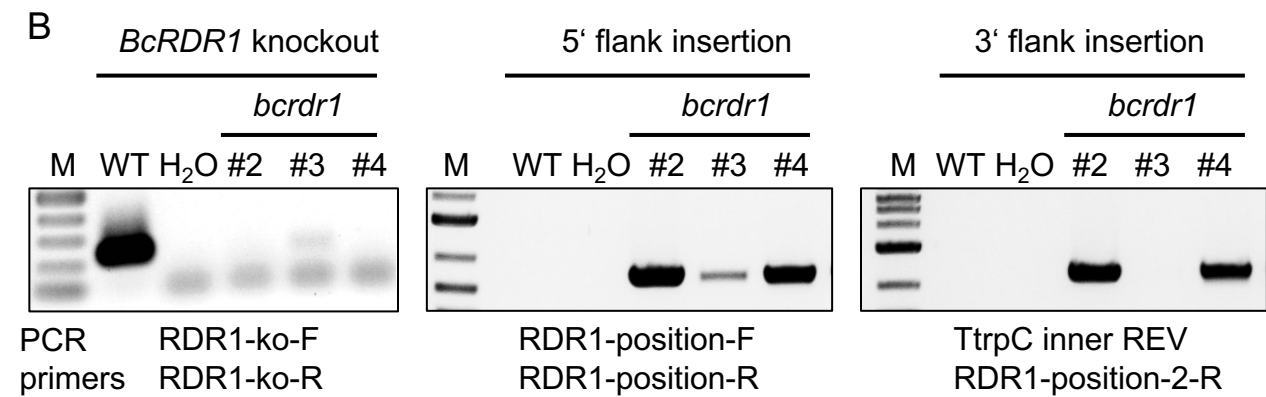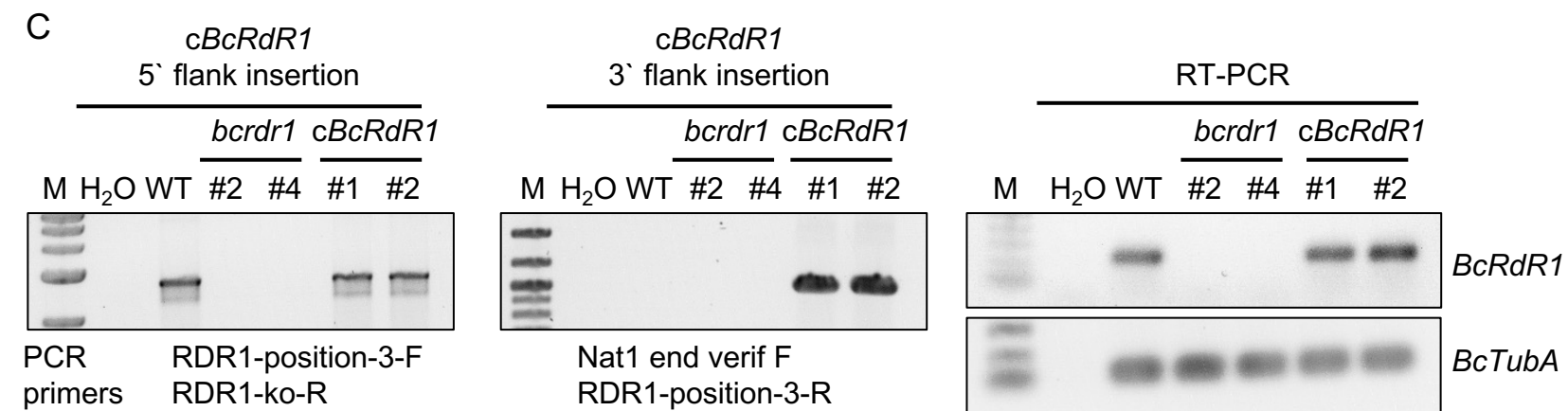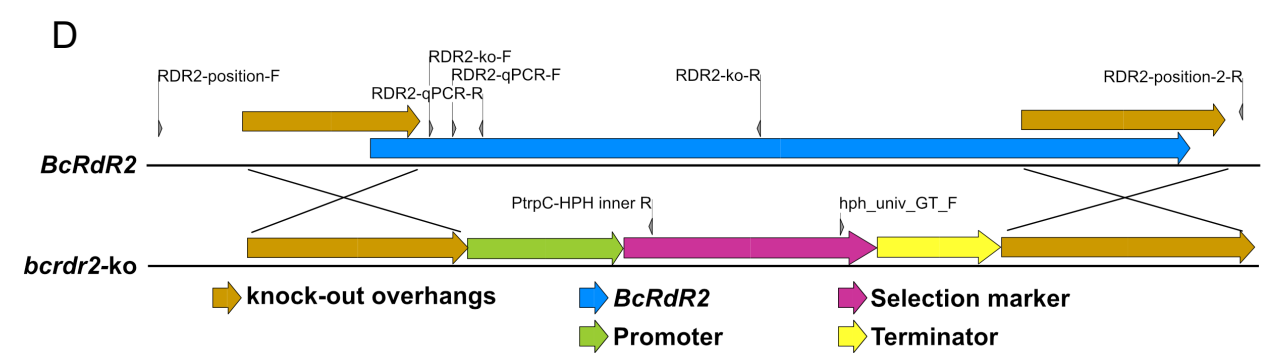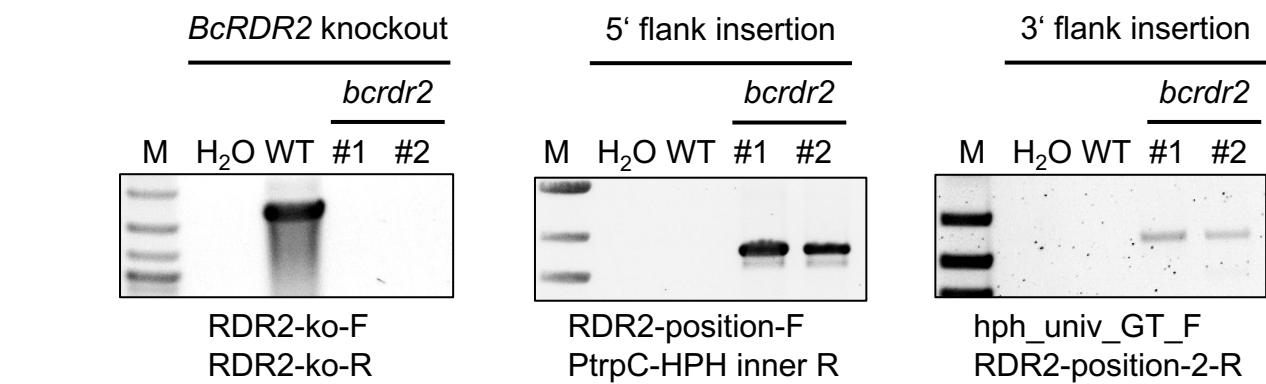

Supplement: S3 Fig — A) Schematic overview of the bcrdr1 ko and cBcRDR1 complementation cloning strategies. B) Genotyping PCRs assessing bcrdr1 gene ko and the insertion of the ko cassette into the BcRDR1 genomic context. C) Genotyping PCRs assessing the insertion of the cBcRDR1 cassette into the bcrdr1 ko genomic context and RT-PCR assessing expression of BcRDR1 in WT, bcrdr1 ko mutant and cBcRDR1 complementation strains. D) Schematic overview of the bcrdr2 ko cloning strategy. E) Genotyping PCR assessing bcrdr2 gene ko. (PDF) [file ppat.1011885.s003.pdf]

A

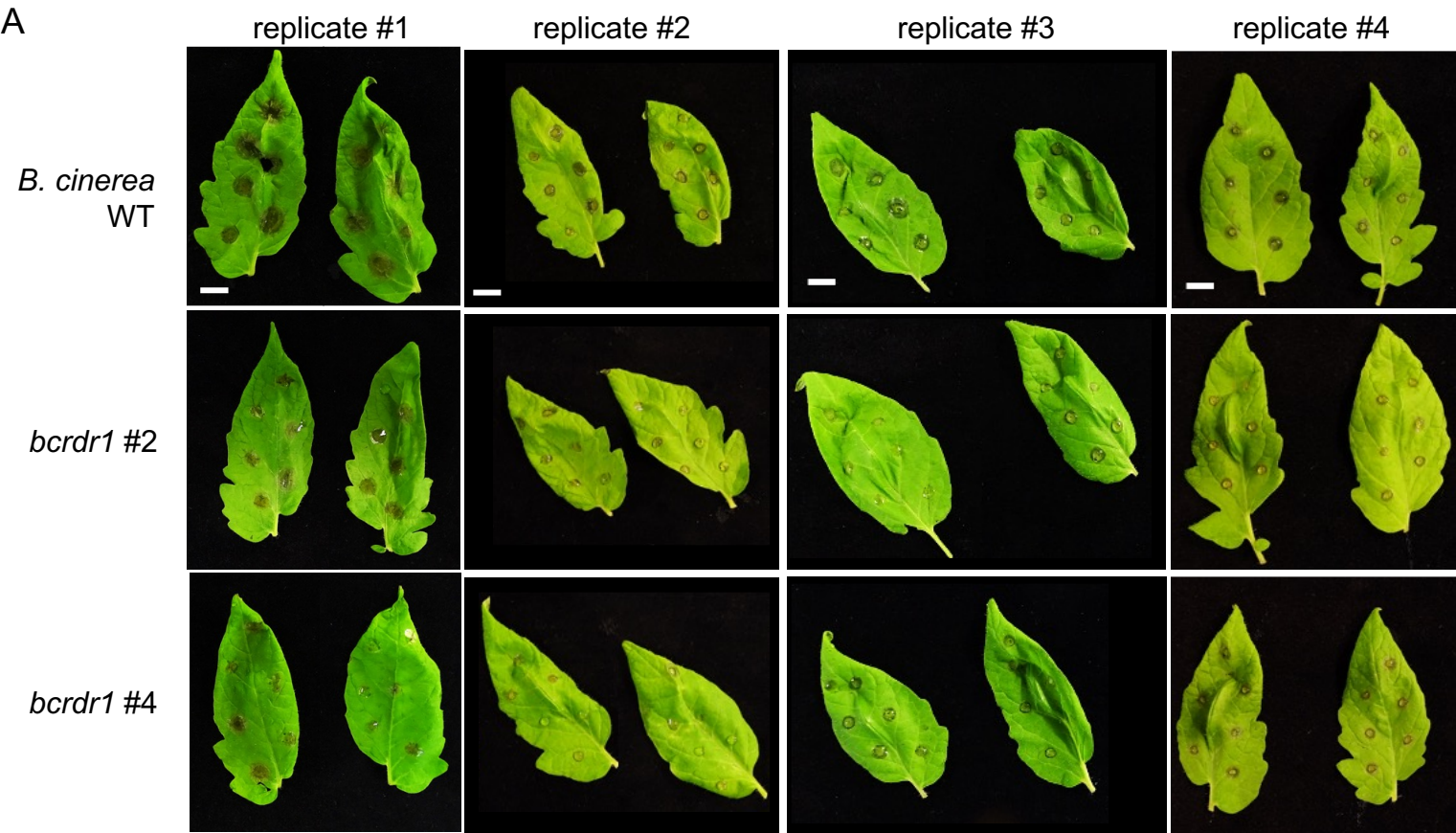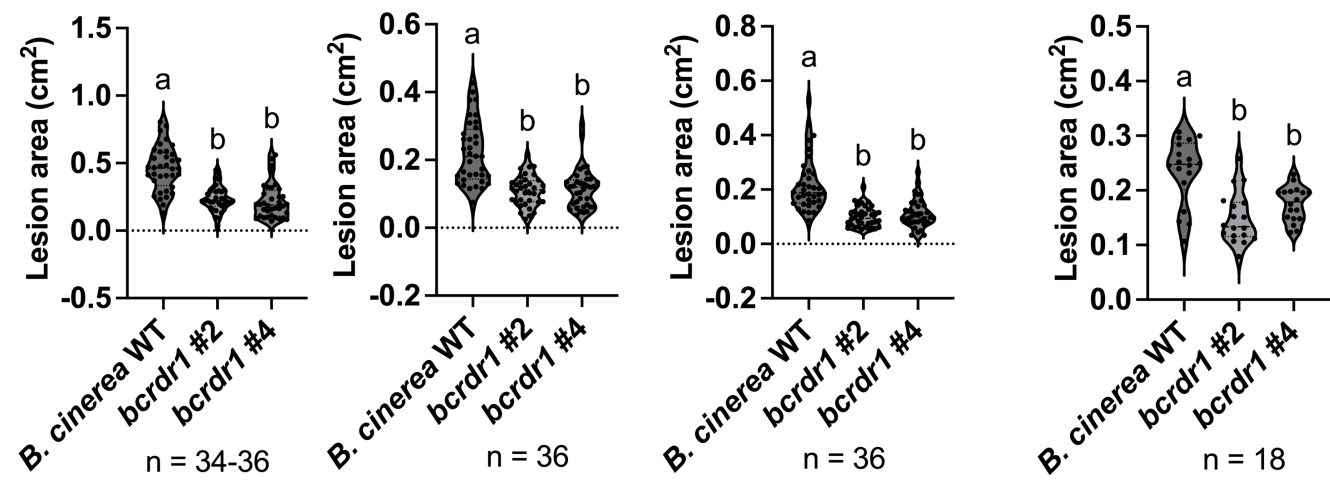

B

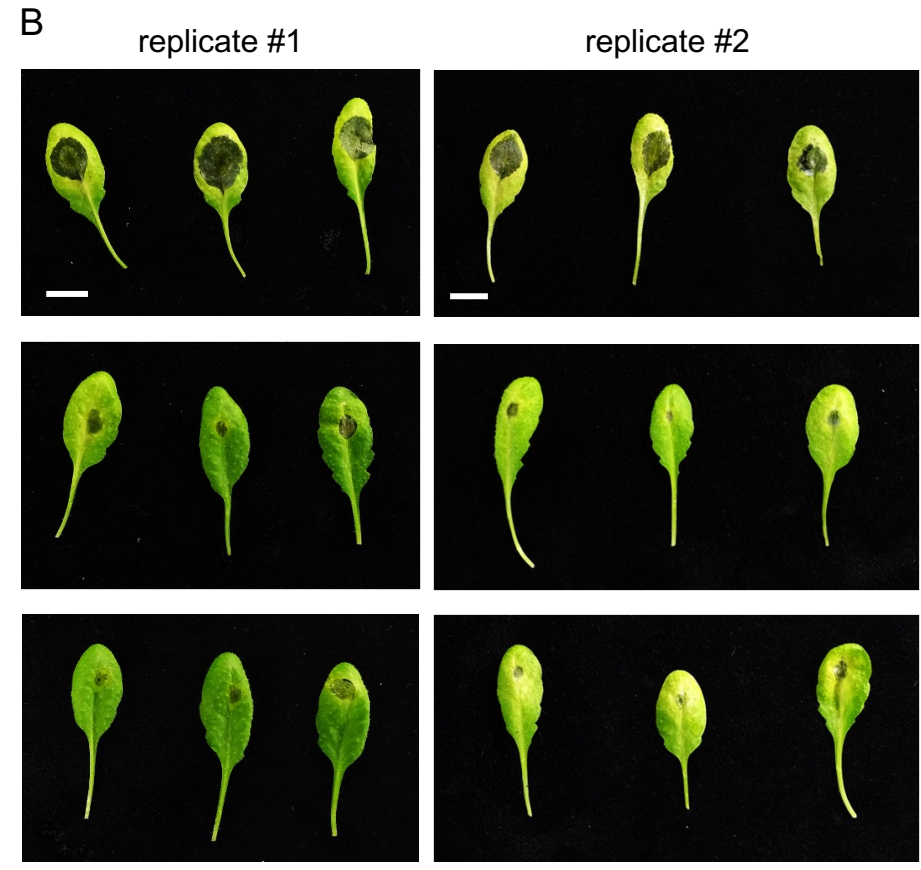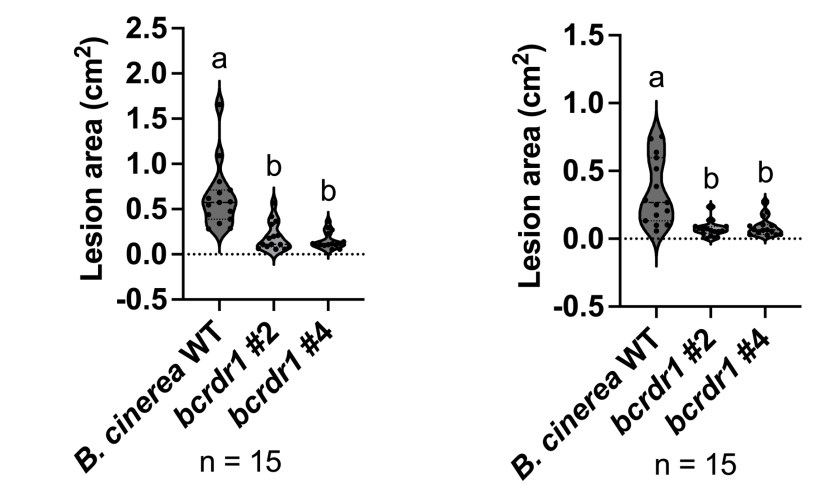

Supplement: S4 Fig — Replicates of infection series with B. cinerea WT and bcrdr1 ko mutants on detached S. lycopersicum (A) and A. thaliana (B) leaves. A 20 μl drop of 5 x 10^4^/ml condidiospores was placed on the leaves. The scale bars represent 1 cm. Lesion size induced by B. cinerea infection was measured at 48 hpi. Numbers given the analyzed lesions per plot. Statistical analysis was performed using ANOVA followed by a Tukey post-hoc test with p-value threshold p < 0.05. (PDF) [file ppat.1011885.s004.pdf]

*BcPG1*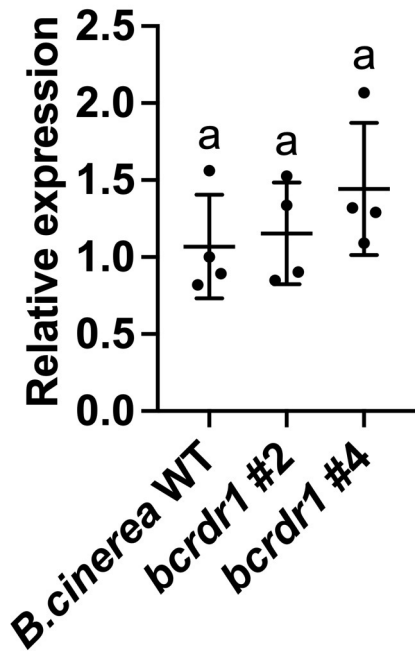*BcNEP1*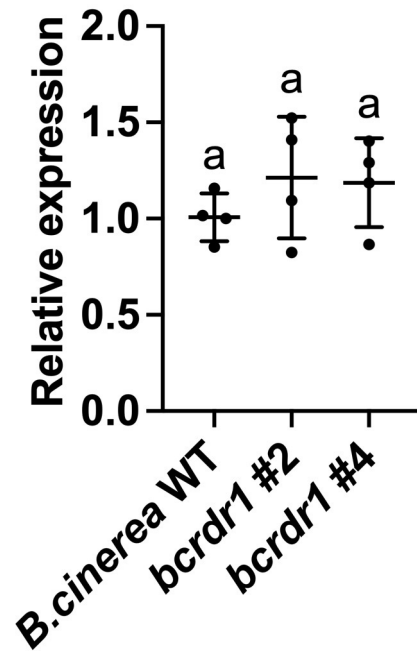*BcSpl1*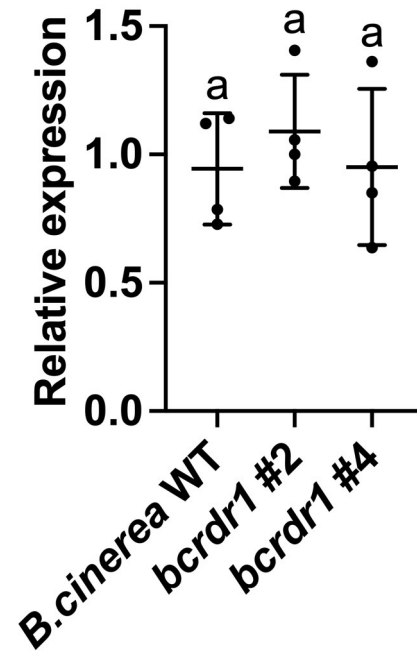*BcXyn11A*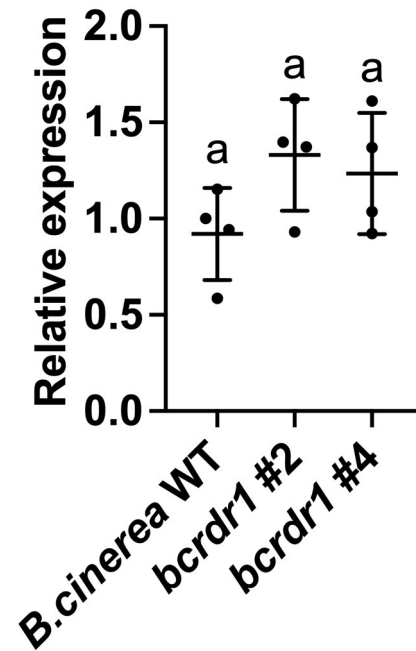*BcHIP1*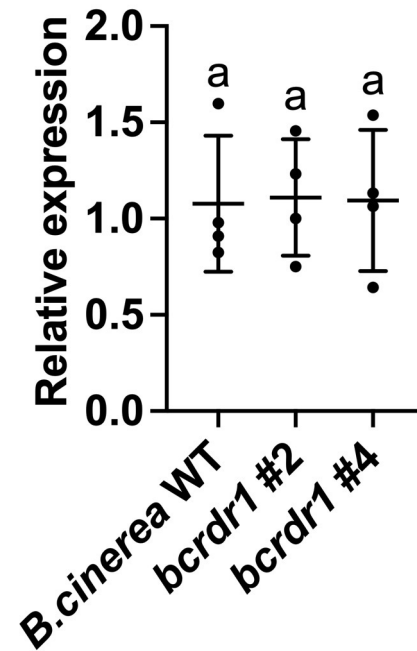

Supplement: S6 Fig — mRNA levels of BcPG1 (Bcin14g00850), BcNEP1 (Bcin06g06720), BcSpl1 (Bcin03g00500), BcXyn11A (Bcin03g00480) and BcHIP1 (Bcin14g01200) was compared in B. cinerea WT and the bcrdr1 ko mutants #2 and #4 grown in axenic culture in four biological replicates. The BcTubA was used as a reference gene. Statistical analysis was performed using ANOVA followed by a Tukey post-hoc test with p-value threshold p < 0.05. (PDF) [file ppat.1011885.s006.pdf]

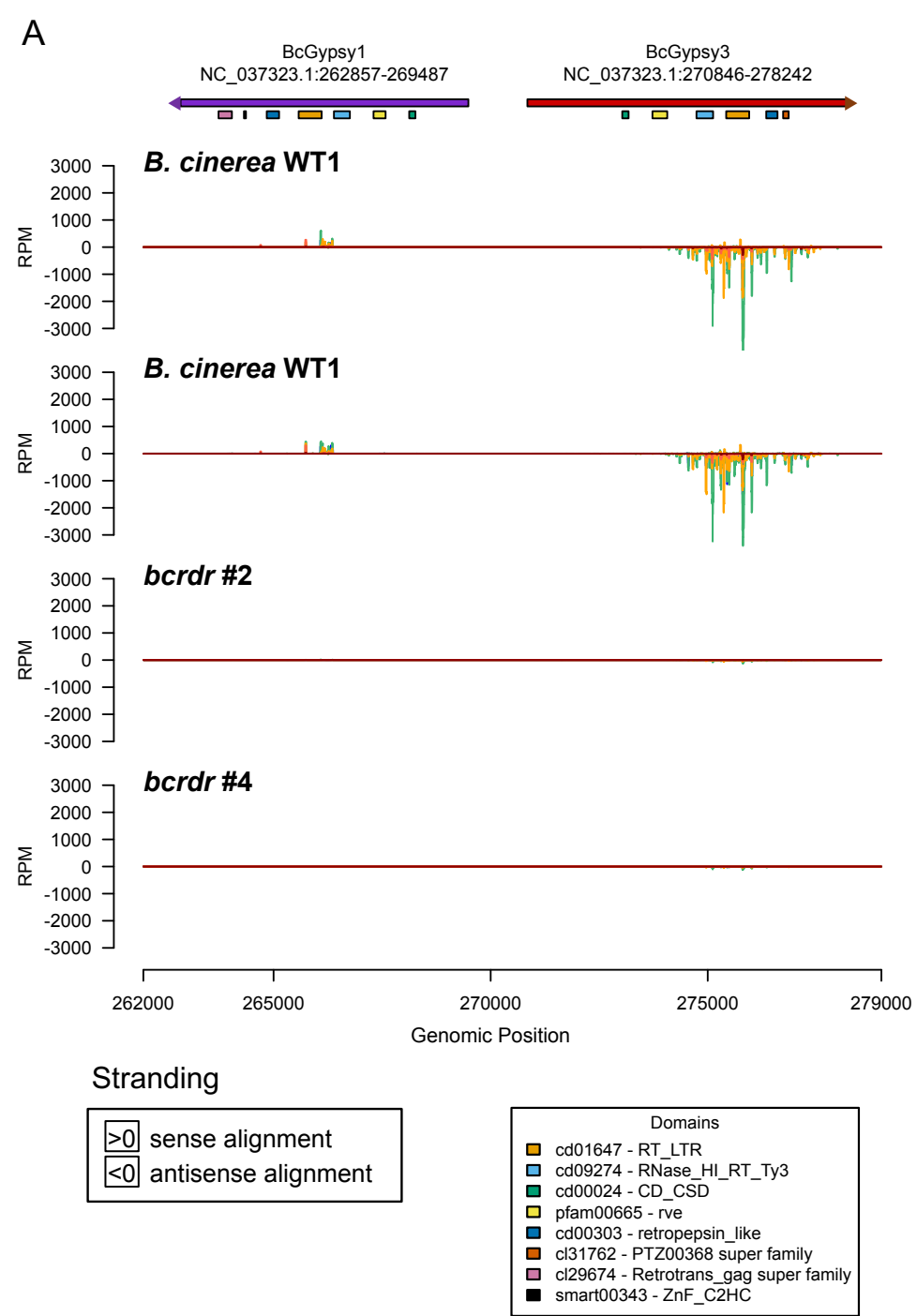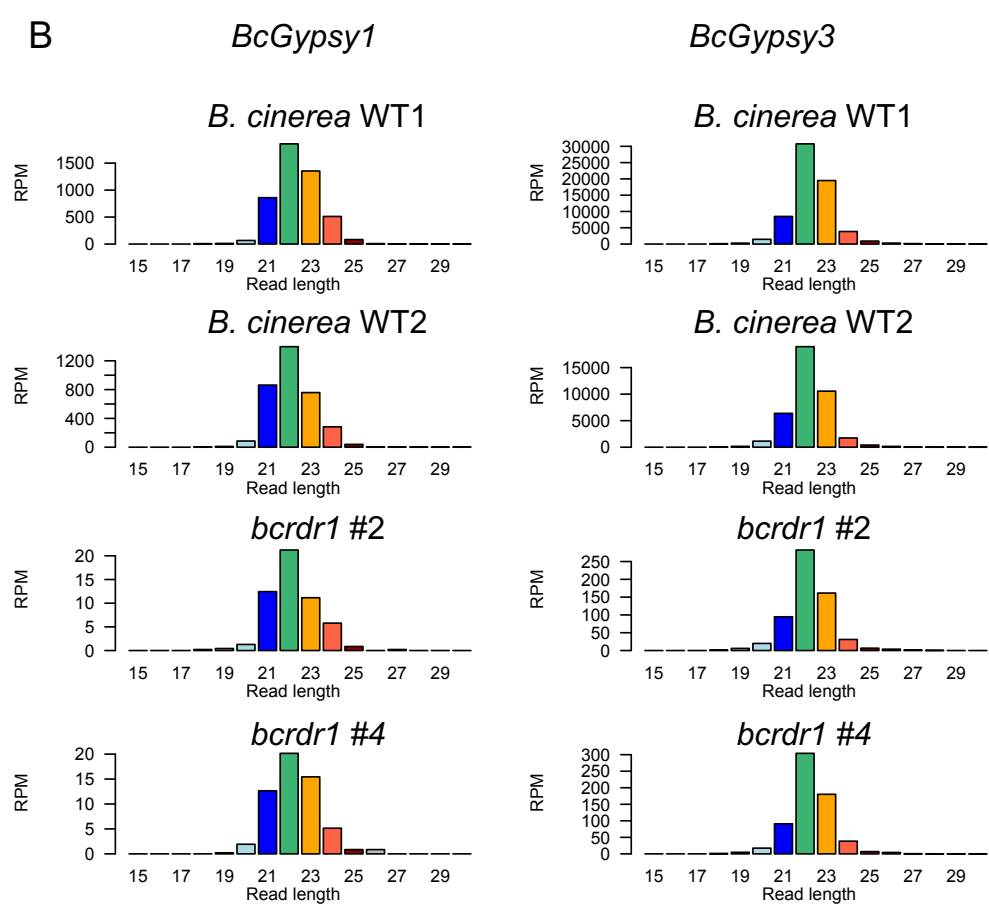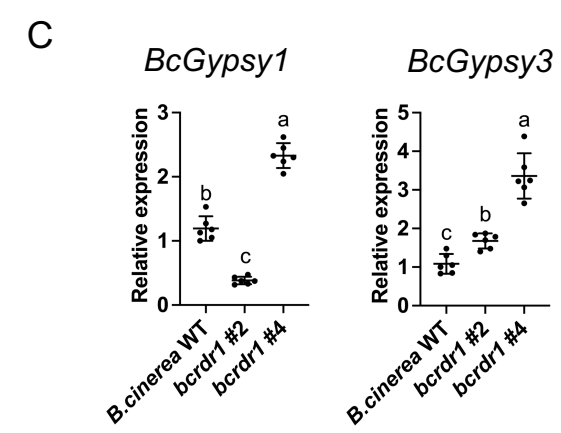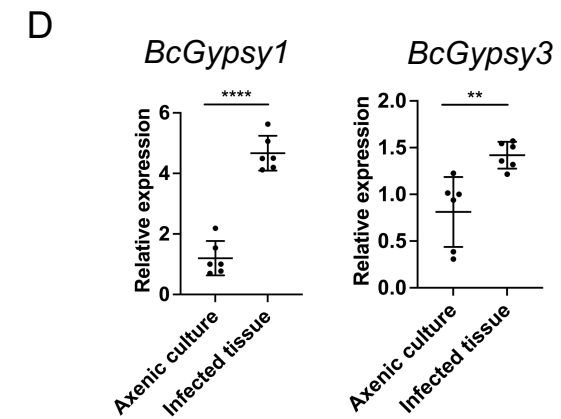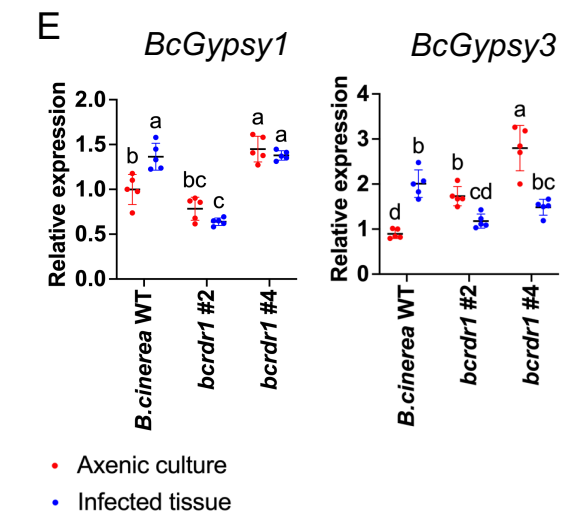

Supplement: S7 Fig — A) Mapping results at the BcGyspy1 and BcGypsy3 loci obtained from B. cinerea WT and bcrdr1 ko mutants small RNA sequencing data. Reads per million (RPM) values > 0 indicate sense alignment, RPM values < 0 indicates antisense alignment. Color-code indicates Bc-sRNA sizes. B) Size profiles of Bc-sRNAs mapped to the BcGypsy1 and BcGypsy3 loci in B. cinerea WT or bcrdr1 ko mutants #2 and #4. C) Expression levels of BcGypsy1 and BcGypsy3 mRNAs in B. cinerea WT and bcrdr1 ko mutants when grown in axenic culture. D) Expression levels of BcGypsy1 and BcGypsy3 mRNAs in B. cinerea WT grown under axenic culture condition and during tomato infection at 48 hpi. E) Expression level comparison of BcGypsy1 and BcGypsy3 mRNAs in B. cinerea WT versus bcrdr1 ko mutants #2 and #4 when grown under axenic culture condition or during tomato infection at 48 hpi. In C), D) and E), BcTubA mRNA was used as a reference gene expression. Lines in scatter plots represent the mean and the standard deviation. Statistical analysis was performed using ANOVA followed by a Tukey post-hoc test with p-value threshold p < 0.05. (PDF) [file ppat.1011885.s007.pdf]

Bc-sRNA3.1

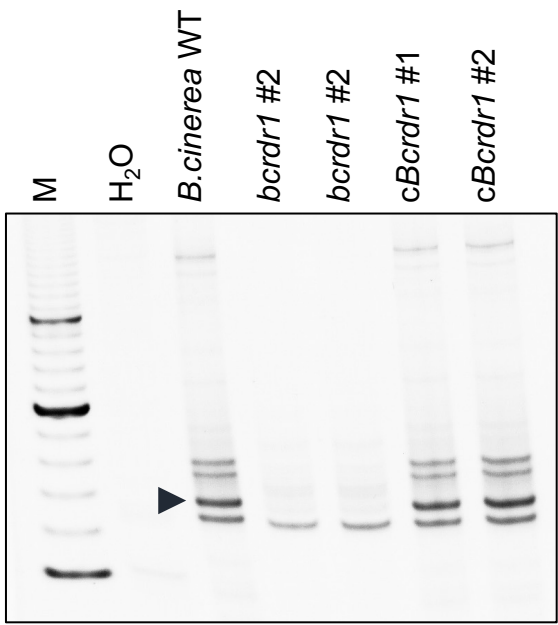

Bc-sRNA3.2

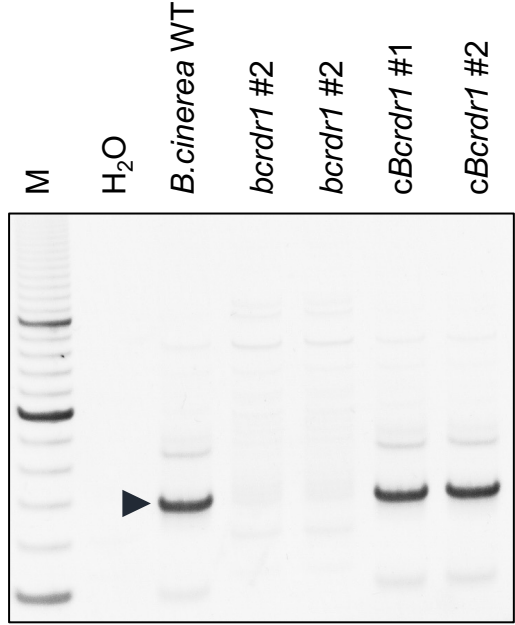

Bc-sRNA5

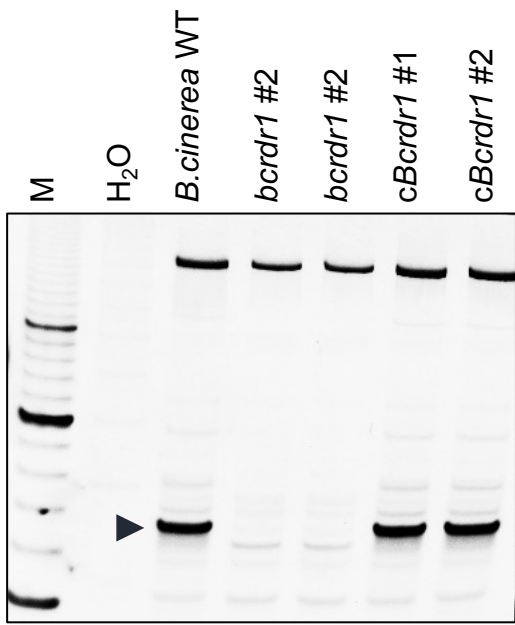

Bc-sRNA20

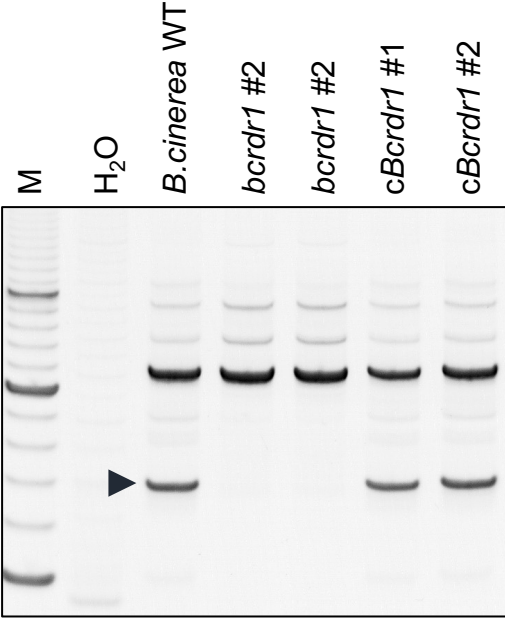

*BcTubA*

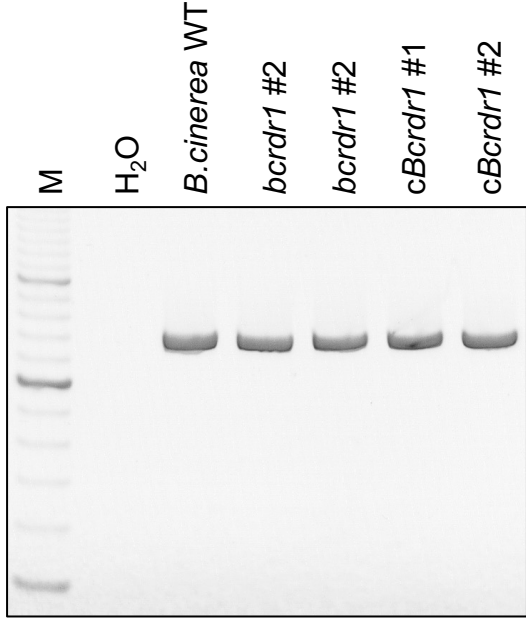

Supplement: S8 Fig — Bc-sRNAs were detected in tomato leaf samples infected with B. cinerea at 48 hpi. Figure represents full-scale gel images of results, as given in Fig 3B. (PDF) [file ppat.1011885.s008.pdf]

A

replicate #1

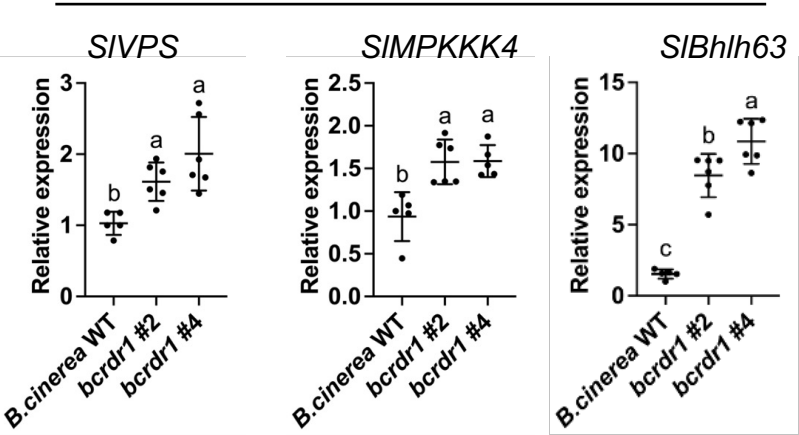

replicate #2

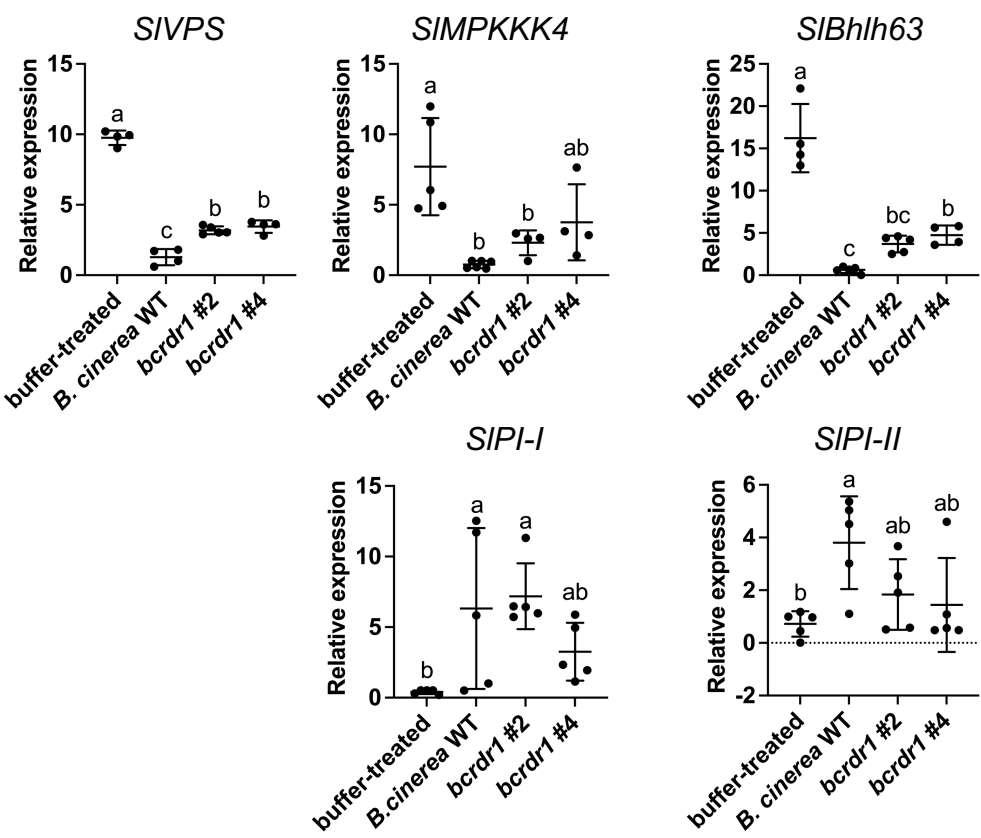

B

replicate #1

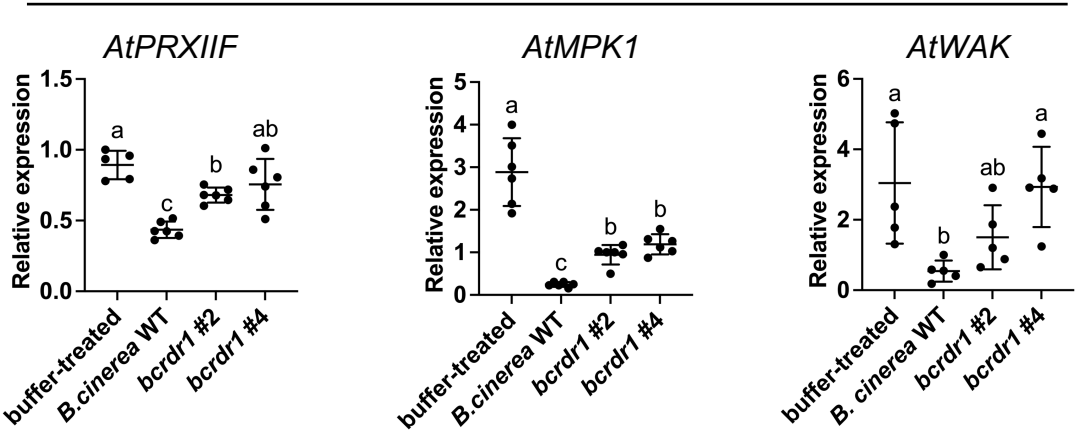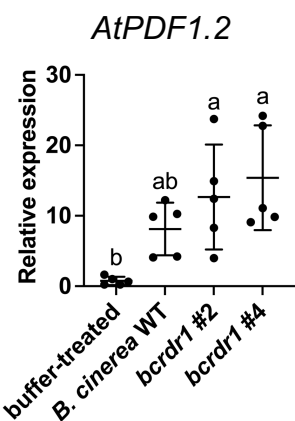

Supplement: S9 Fig — Biological replicates of mRNA expression measurements of known Bc-sRNA target genes in S. lycopersicum (A) and A. thaliana (B) during infection with B. cinerea WT and bcrdr1 ko mutants. Samples were taken at 36 hpi or 60 hpi for S. lycopersicum and A. thaliana, respectively. The SlActin2 or the AtActin2 were used as reference genes. Lines in scatter plots represent the mean and the standard deviation. Statistical analysis was performed using ANOVA followed by a Tukey post-hoc test with p-value threshold p < 0.05. (PDF) [file ppat.1011885.s009.pdf]

A

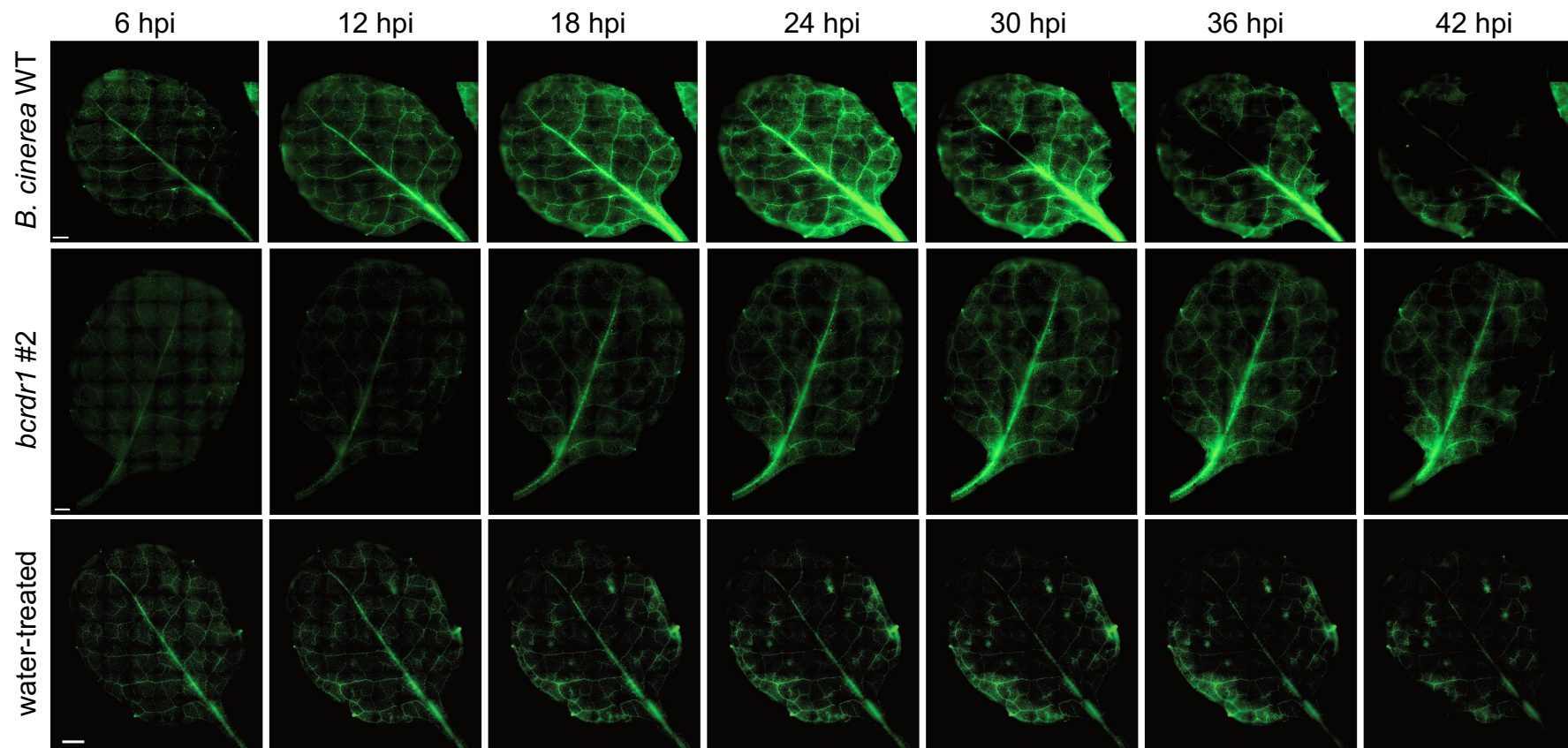

B

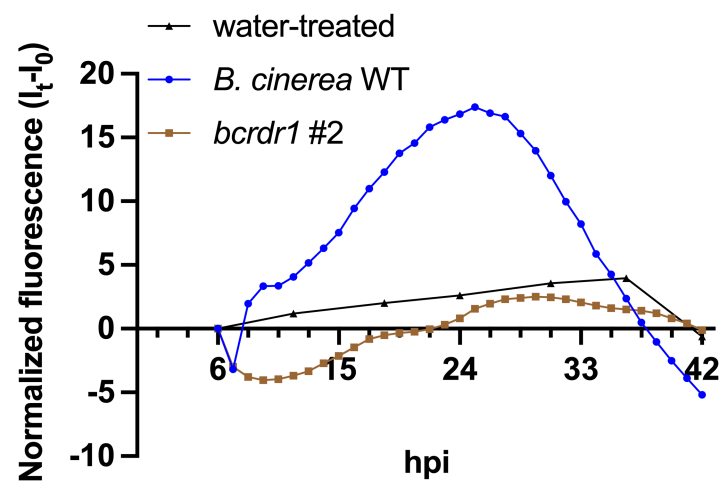

Supplement: S10 Fig — A) Fluorescence microscopy images at different time points of infection. A 5 μl drop of a 2 x 10^5^/ml conidiospore suspension was placed at the center of the leaf before placing a glass covering slip on the top that dispersed the spore suspension and led to GFP activation over the entire leaf. The scale bars represent 1 mm. B) Normalized GFP signal quantification of whole seedling leaves over the time series of 6–42 hpi. (PDF) [file ppat.1011885.s010.pdf]

*A. thaliana* WT infected with *B. cinerea* WT

0 hpi

6 hpi

12 hpi

18 hpi

24 hpi

30 hpi

36 hpi

42 hpi

48 hpi

Bright Field

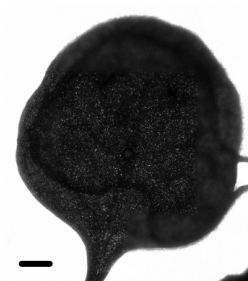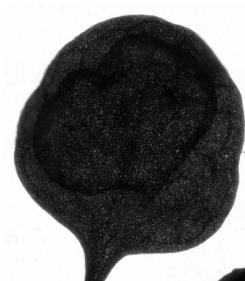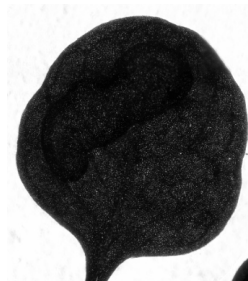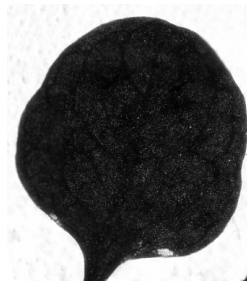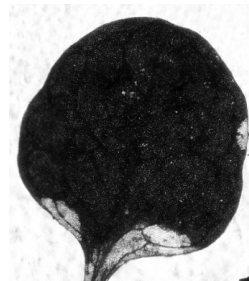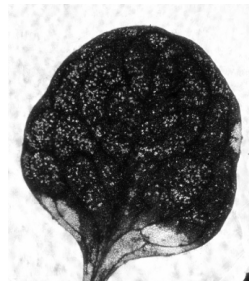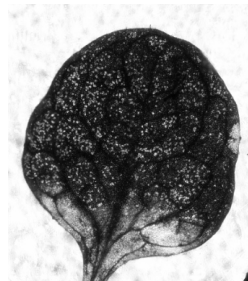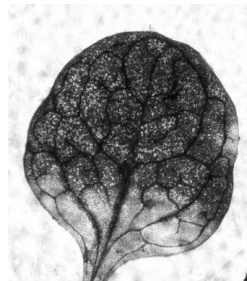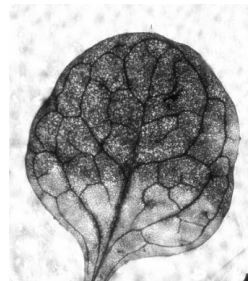

GFP

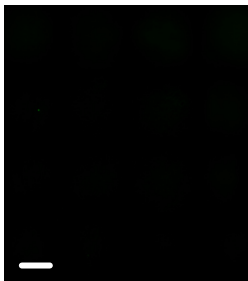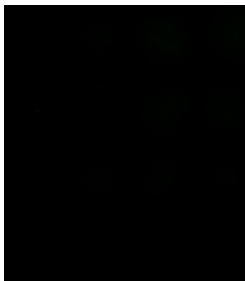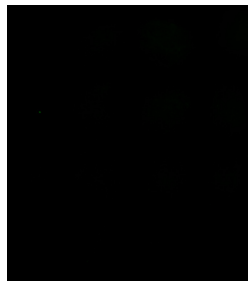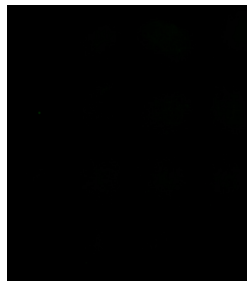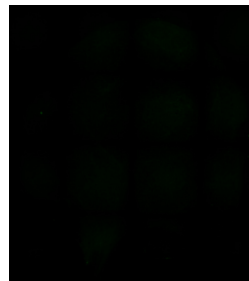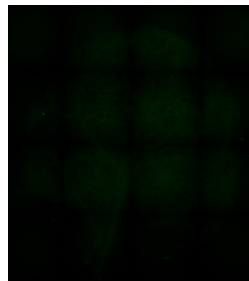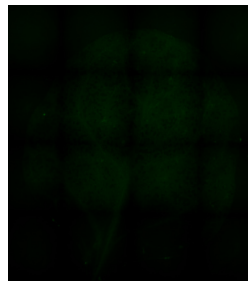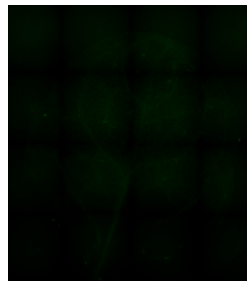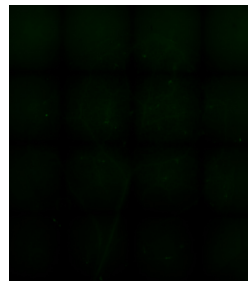

Supplement: S11 Fig — The scale bars represent 1 mm. (PDF) [file ppat.1011885.s011.pdf]

Infection series #1

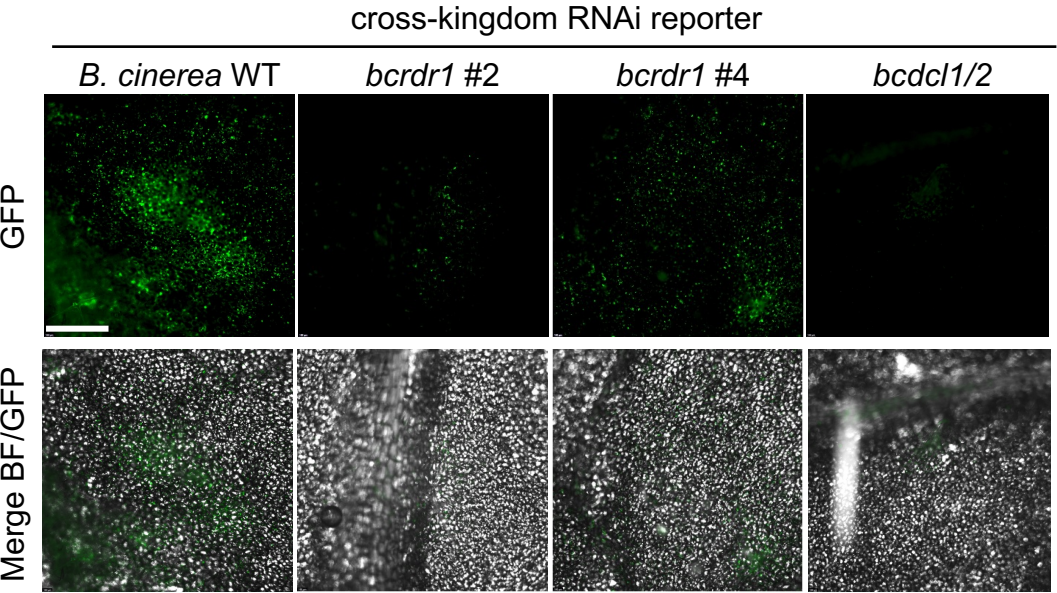

Infection series #2

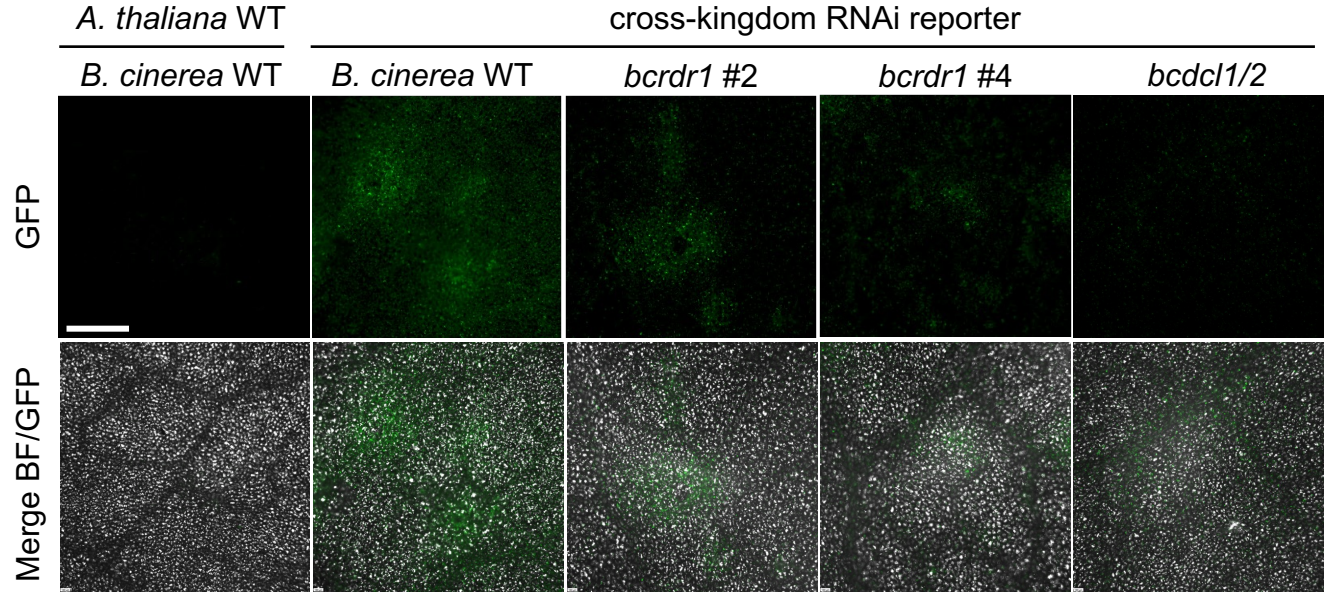

Infection series #3

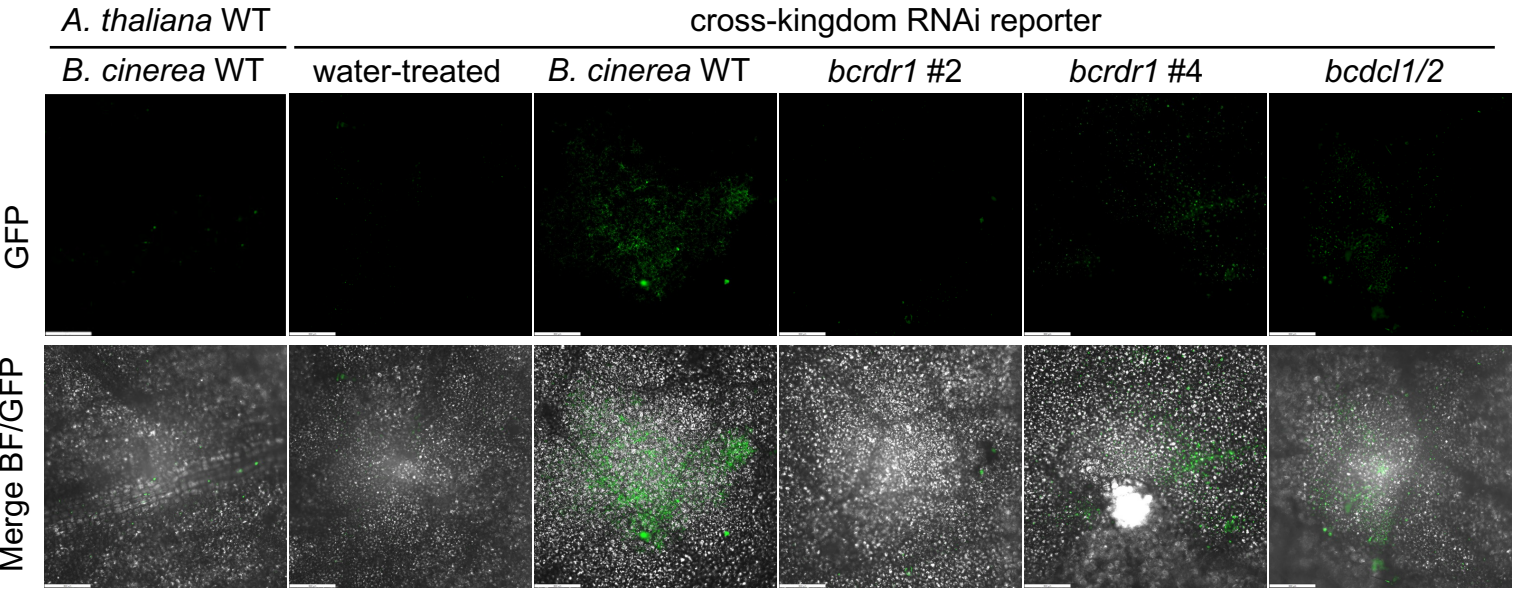

Supplement: S12 Fig — Leaves were inoculated with a 20 μl drop of 2 x 10^5^/ml conidiospore suspension of B. cinerea WT, bcrdr1 ko mutants, and a bcdcl1/2 mutant. A. thaliana WT plants were infected with B. cinerea WT to assess auto-fluorescence, and water-treated GFP reporter plants were assessed for reporter auto-activity. The scale bars represent 500 μm. (PDF) [file ppat.1011885.s012.pdf]

GFP

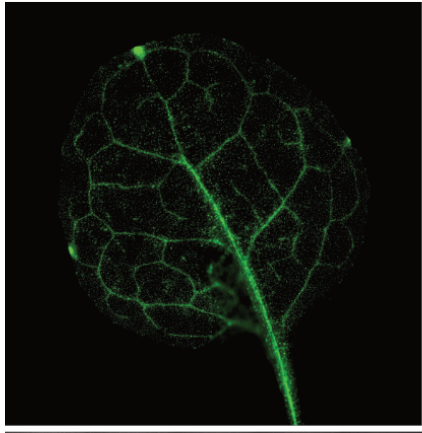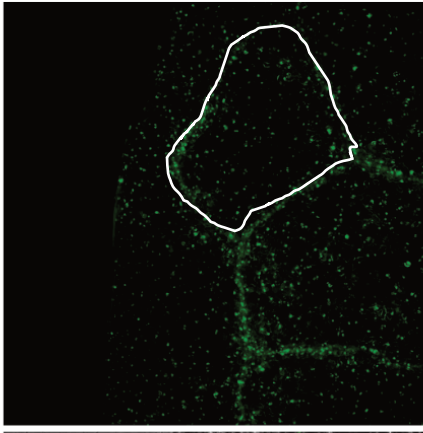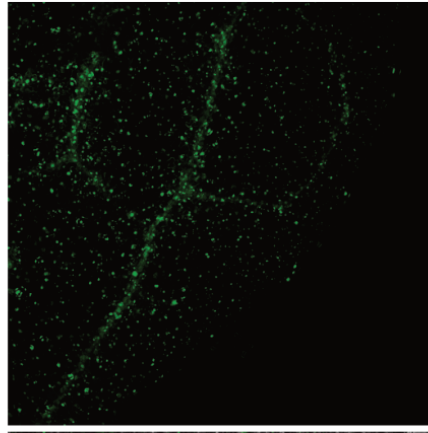

Merged BF/GFP

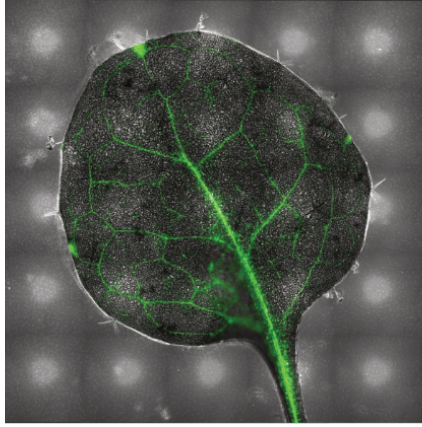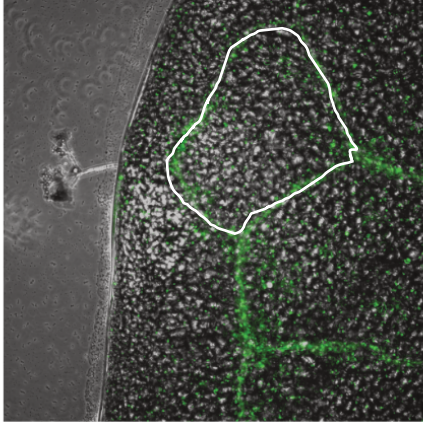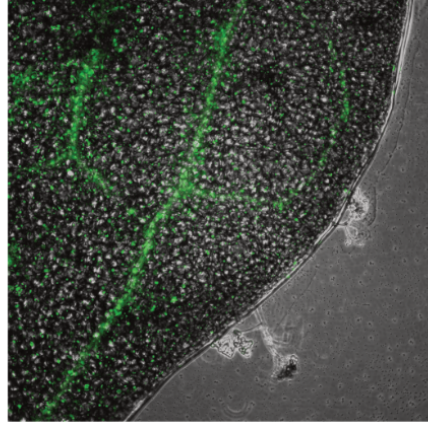

Trypan blue

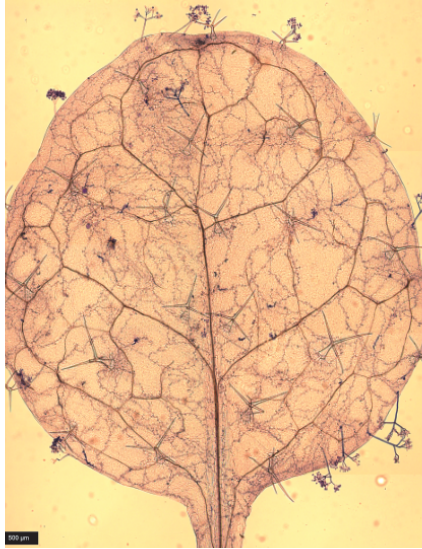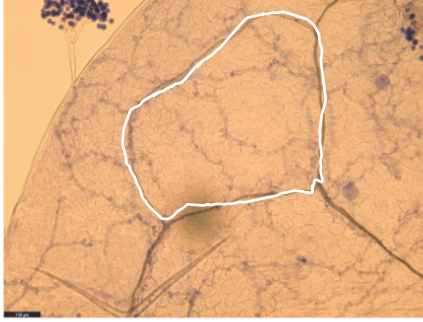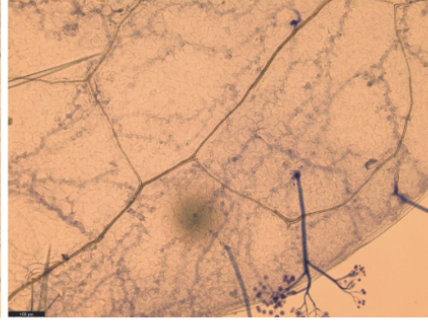

Supplement: S13 Fig — 10 ml of a 2 x 10^4^/ml conidiospore suspension was sprayed onto leaves. Trypan Blue staining visualized oomycete hyphae in the infected leaf. Outlines indicate the same leaf area in fluorescence and Trypan Blue staining images. (PDF) [file ppat.1011885.s013.pdf]

*H. arabidopsidis*

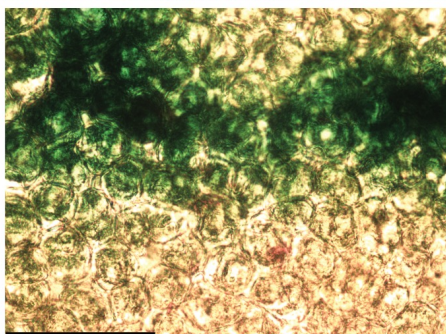

*B. cinerea*

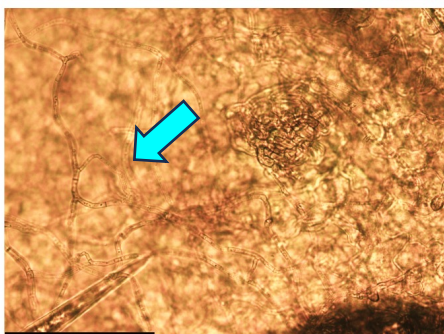

*H. arabidopsidis*

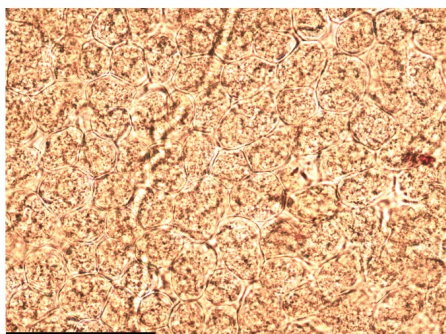

*B. cinerea*

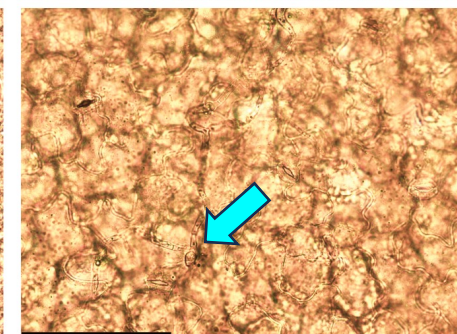

Supplement: S14 Fig — This reporter line was previously designed to demonstrate cross-kingdom RNAi triggered by small RNAs secreted by the oomycete H. arabidopsidis (Dunker et al., 2020 [22]). A) Infection of the GUS reporter line with H. arabidopsidis showing GUS at infecting oomycete hyphae. B) Infection of the GUS reporter line with B. cinerea revealed no GUS activation at infection sites (indicated by a turquoise arrows). C) Infection of a GUS reporter line carrying scrambled small RNA target sites with H. arabidopsidis showing no GUS activity. D) Infection of the scrambled GUS reporter line with B. cinerea showing no GUS activity at infection sites (indicated by a turquoise arrows). For B. cinerea inoculation, a 20 μl drop of 2 x 10^5^ conidiospores were placed onto leaves. For H. arabidopsidis infection, 10 ml of a 2 x 10^4^/ml conidiospore suspension were sprayed onto leaves. The scale bars represent 100 μm. (PDF) [file ppat.1011885.s014.pdf]

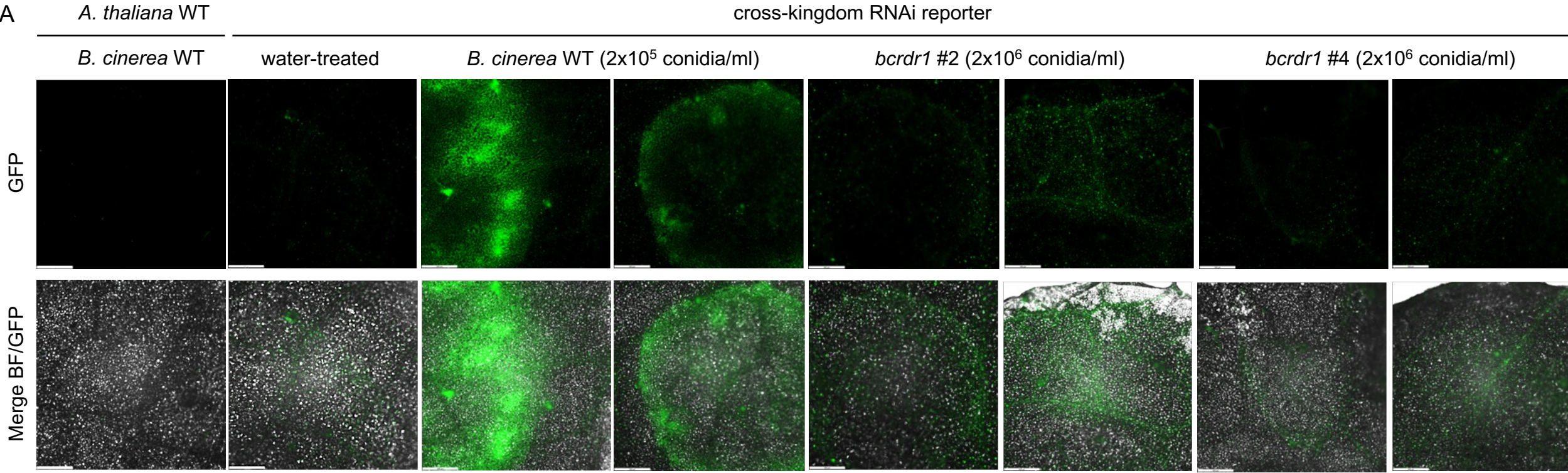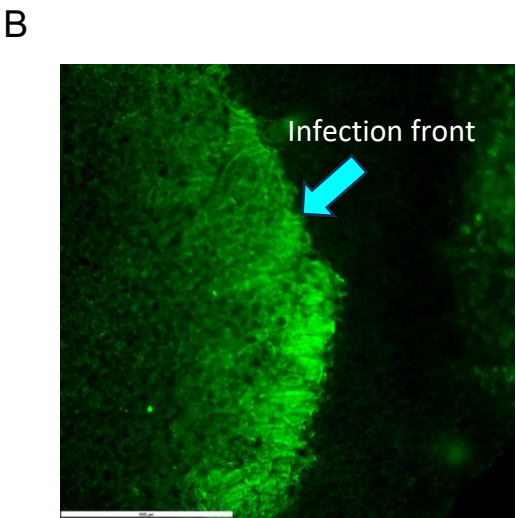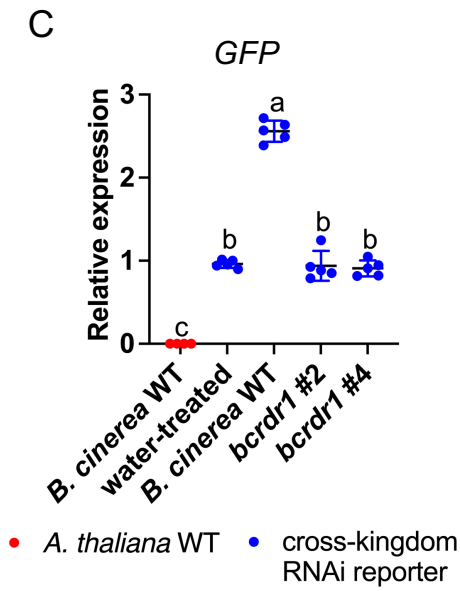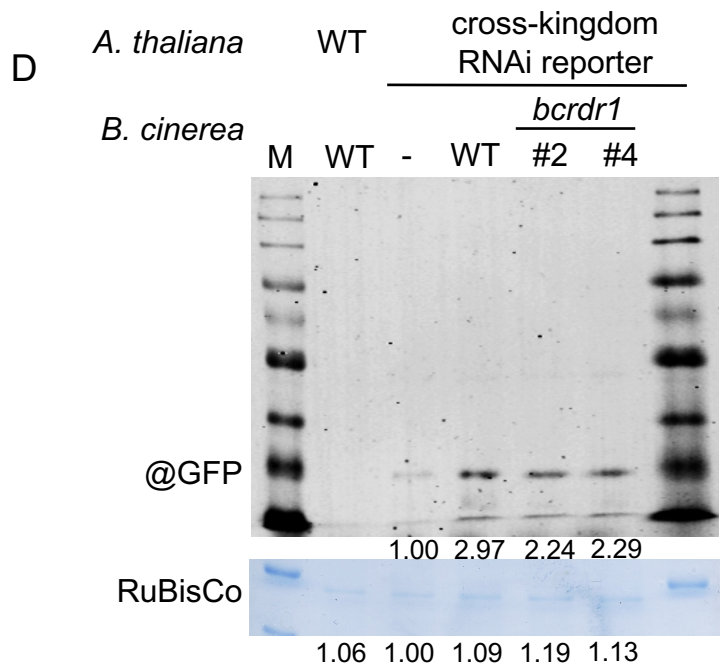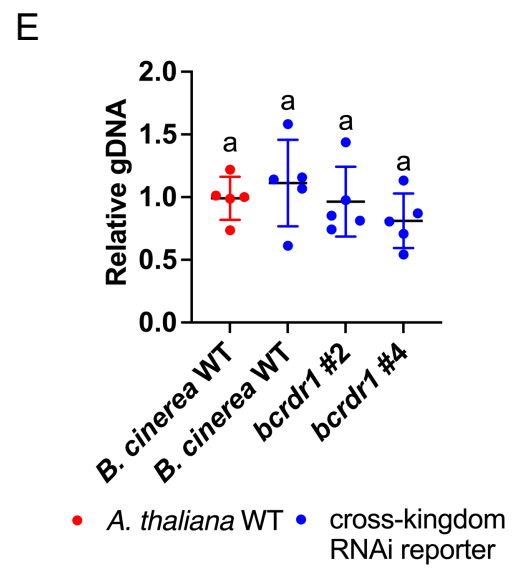

Supplement: S15 Fig — A) Fluorescence microscopy images at 24 hpi indicated enhanced GFP expression in reporter plants at infection sites of B. cinerea WT in contrast to GFP non-expressing A. thaliana WT plants, water-treated GFP reporter plants or GFP reporter plants infected with bcrdr1 ko mutants. The turquoise arrow in B) indicates the infection front of the B. cinerea WT inoculation. The scale bars in A) and B) represent 500 μm. C) GFP mRNA expression levels in A. thaliana WT plants (red dots), A. thaliana GFP reporter plants (blue dots) infected with B. cinerea WT, bcrdr1 ko mutants or water-treated. The AtActin2 was used as a reference gene. D) Western blot analysis of GFP expression using a @GFP antibody. RuBisCo signals were visualized by Coomassie Brilliant Blue staining. Numbers indicate GFP and RuBisCo intensities estimated by the FIJI software. E) B. cinerea genomic DNA in infected A. thaliana WT or GFP reporter plants was measured by qPCR using primers of the BcTubA gene. Raw data were normalized to plant DNA using AtActin2 primers. Lines in scatter plots of qRT-PCR data in C) and qPCR of B. cinerea genomic DNA in E) represent the mean and the standard deviation. Statistical analysis was performed using ANOVA followed by a Tukey post-hoc test with p-value threshold p < 0.05. (PDF) [file ppat.1011885.s015.pdf]

GFP

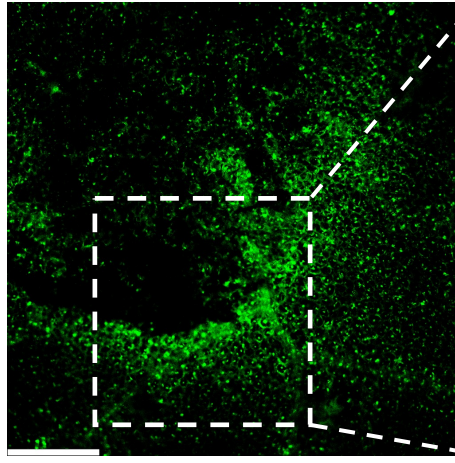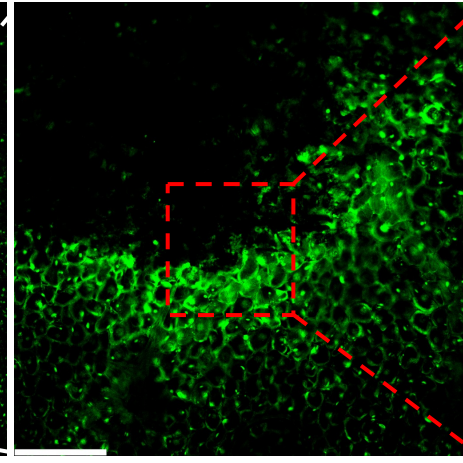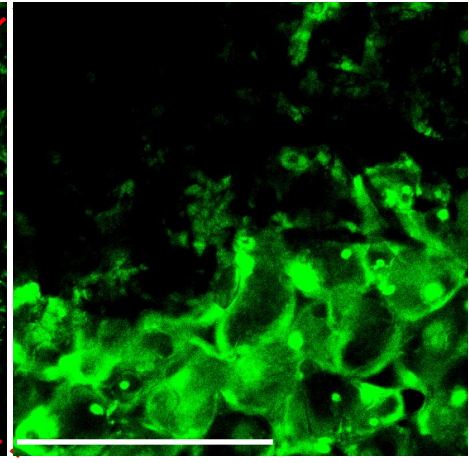

Merged BF/GFP

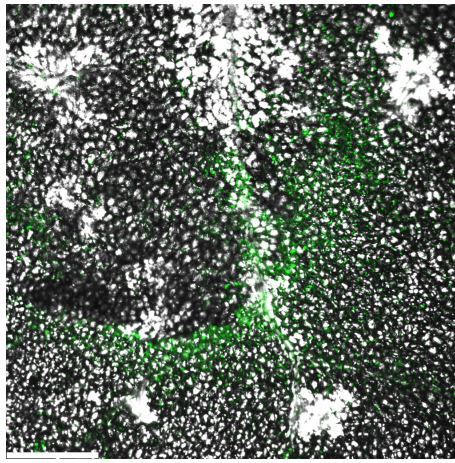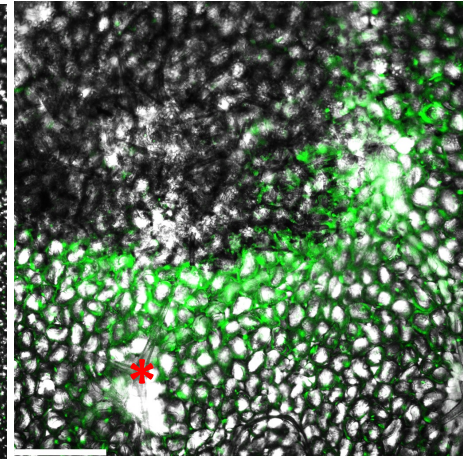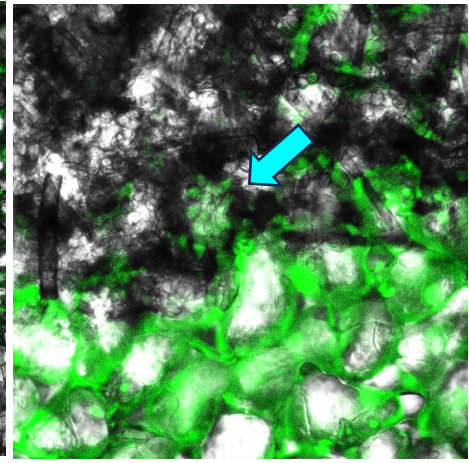

Trypan blue

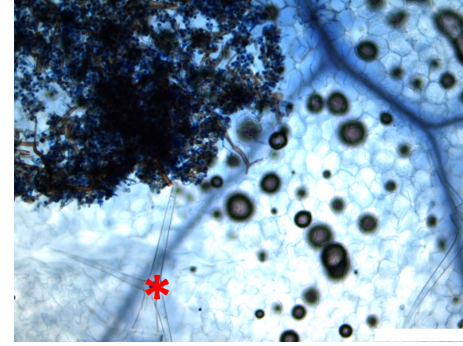

Supplement: S16 Fig — B. cinerea mycelium was visualized by Trypan Blue staining. Squares in images indicate area of magnification. The red asterisks indicate the same leaf trichome in merged BF/GFP and Trypan Blue images. The turquoise arrow indicates B. cinerea mycelium. The scale bars represent 500 μm. (PDF) [file ppat.1011885.s016.pdf]

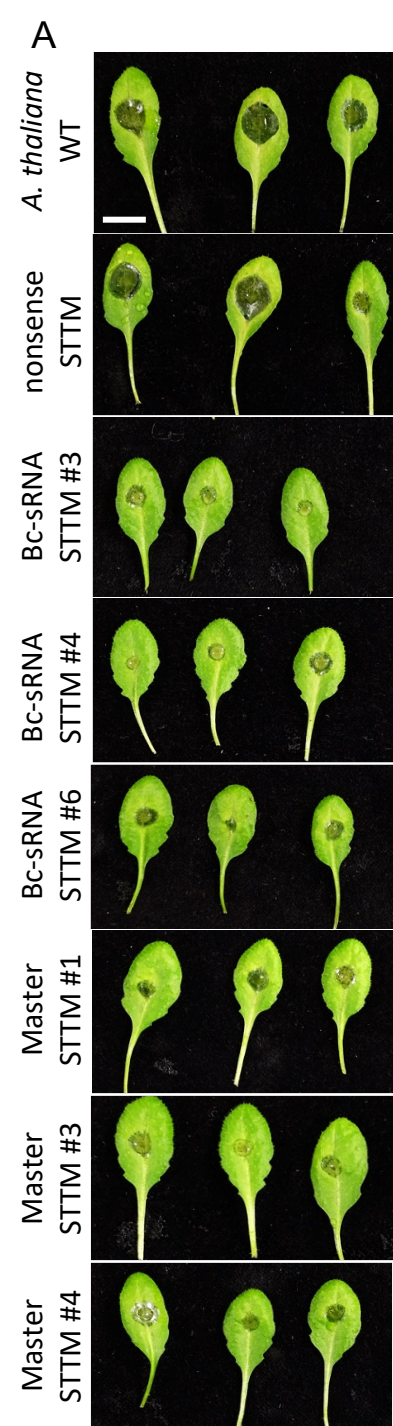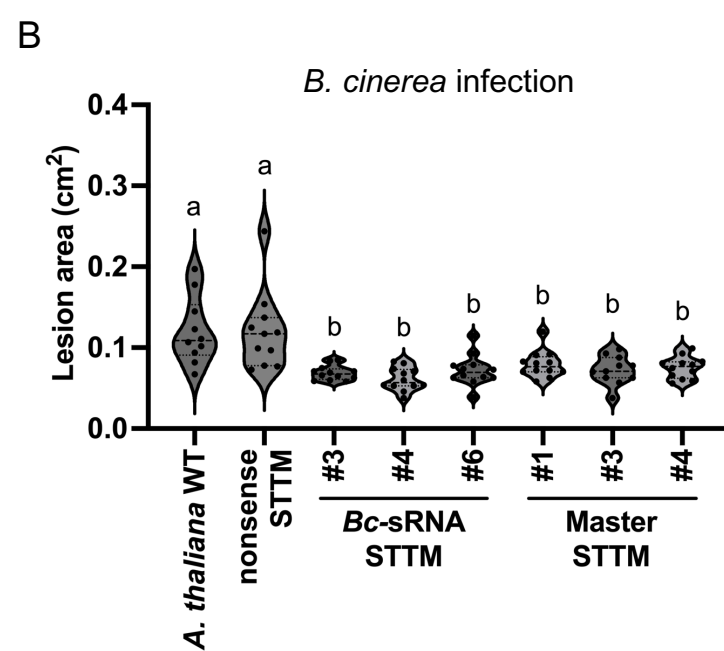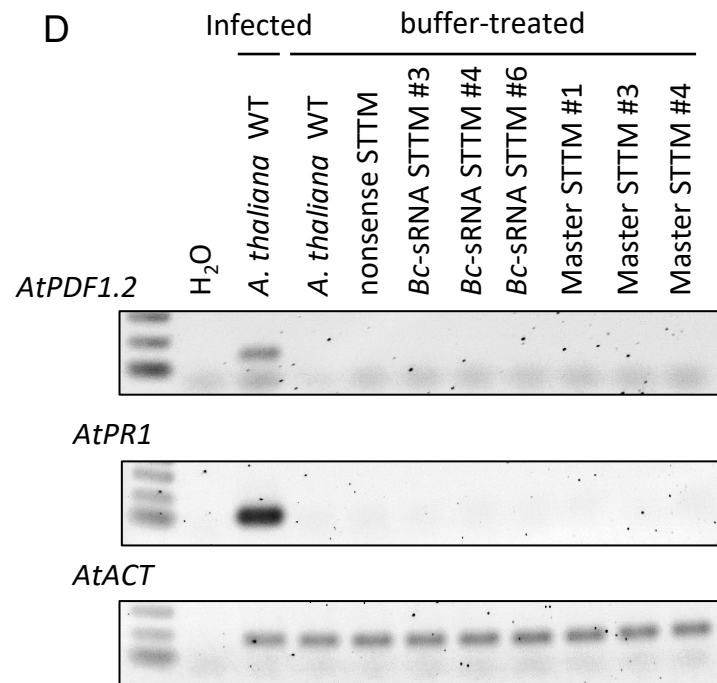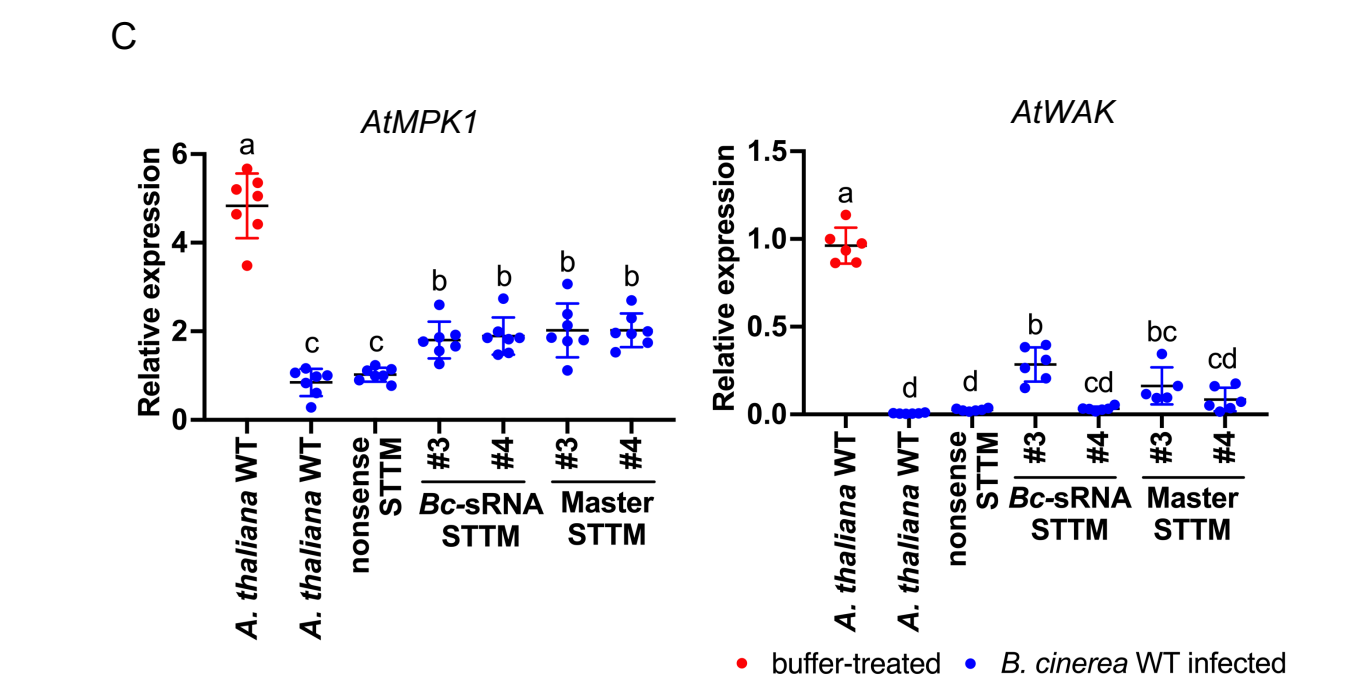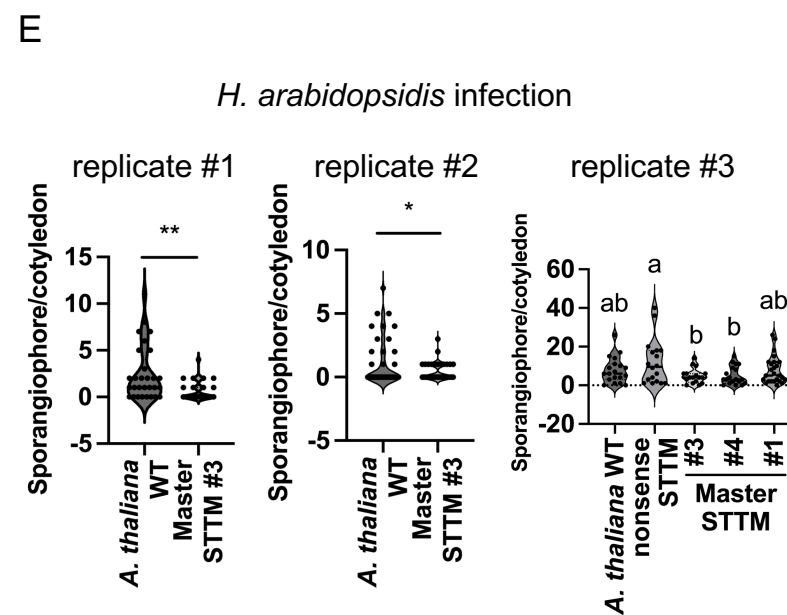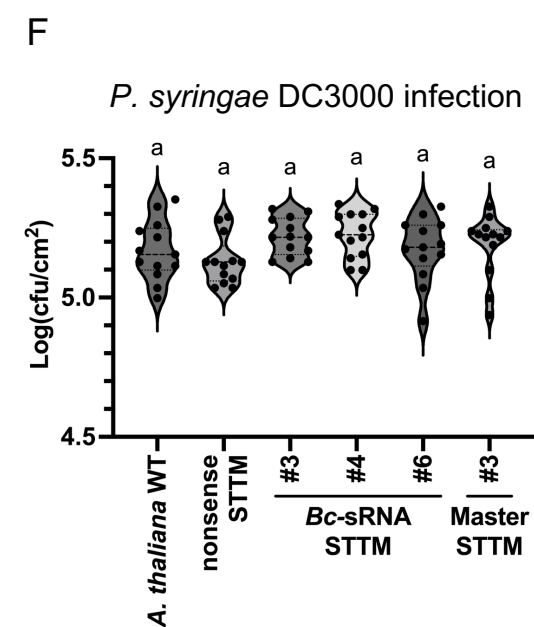

Supplement: S17 Fig — A) Leaf images of A. thaliana STTMs upon B. cinerea infection with 2 x 10^5^ conidiospores/ml at 60 hpi. The scale bar in represent 1 cm. B) Lesion area induced by B. cinerea infection was measured at 48 hpi. C) mRNA expression levels of Bc-sRNA target genes AtMPK1 and AtWAK in A. thaliana. The AtActin2 was used as a reference gene. Lines in scatter plots represent the mean and the standard deviation. Statistical analysis was performed using ANOVA followed by a Tukey post-hoc test with p-value threshold p < 0.05. D) Semi-quantitative RT-PCR of the A. thaliana immunity-associated genes AtPR1 and AtPDF1.2. B. cinerea-infected leaves were used as an immunogenic control. E) Infection of the master STTM line #3 with the oomycete H. arabidopsidis. Oomycete sporangiophores were counted at 7 dpi in three replicated inoculation experiments. F) Infection of A. thaliana STTM lines with the bacterial pathogen Pseudomonas syringae DC3000. Colony-forming units (cfu) of were counted at 3 dpi. Statistical analysis in B), C), E), F) was performed using ANOVA followed by a Tukey post-hoc test with p-value threshold p < 0.05. Statistical analysis in E) replicate #1 and replicate #2 was carried out by unpaired t-test with two-tailed p-value < 0.05 (*), p-value < 0.01 (**). (PDF) [file ppat.1011885.s017.pdf]
