# Supplementary material for: A fungal RNA-dependent RNA polymerase is a novel player in plant infection and cross-kingdom RNA interference
Source: PLoS Pathog. 2023 Dec 20;19(12):e1011885. doi: 10.1371/journal.ppat.1011885 (PMC10766185; doi:10.1371/journal.ppat.1011885)
Supplement: S5 Fig — A) Plate growth images were taken at 4 days. The scale bars represent 1 cm. B) To measure growth curves, a drop of 2 x 10^5^/ml 10 μl conidiospore suspension was placed at the center of the agar plate and colony diameter was measured at 3, 4 and 5 days. Data represent 5 replicates. Statistical analysis was performed using ANOVA followed by a Tukey post-hoc test with p-value threshold p < 0.05. (PDF) [file ppat.1011885.s005.pdf]

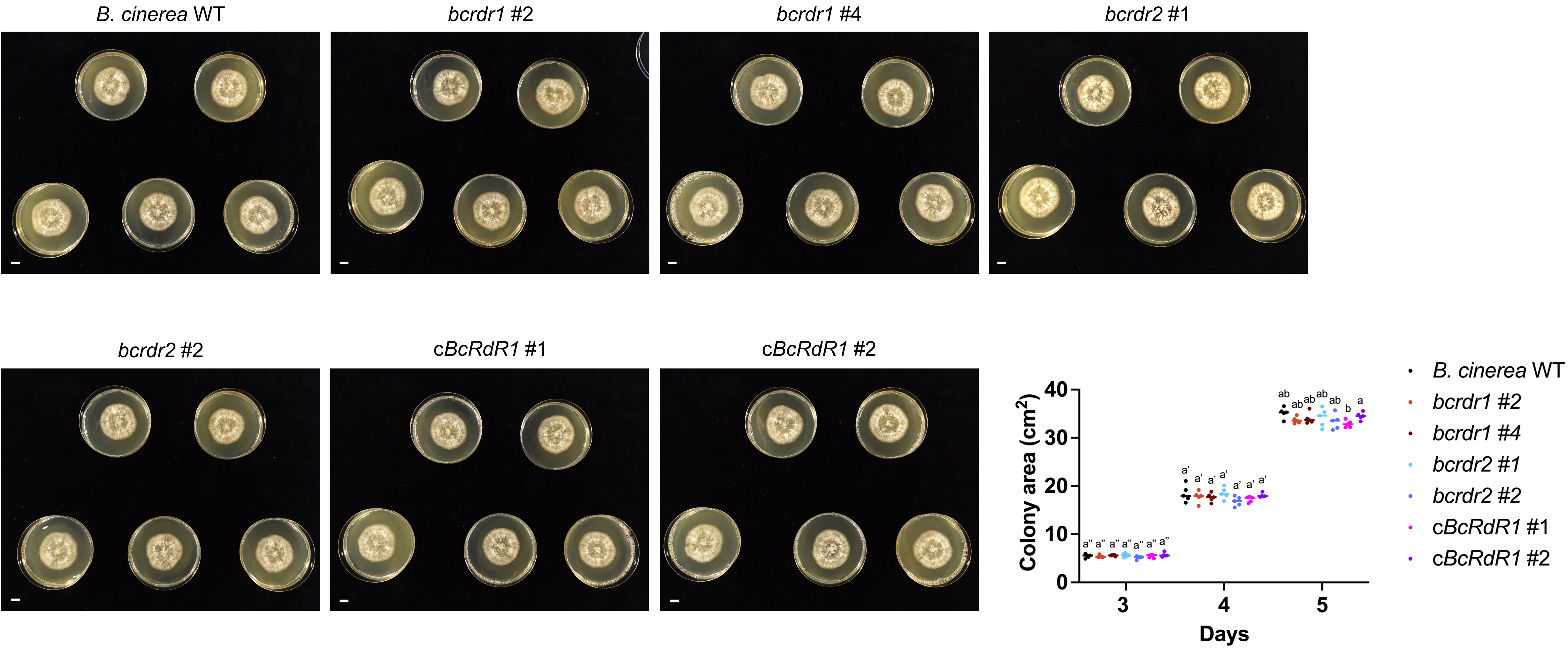
B. cinerea WT
bcrdr1 #2
bcrdr1 #4
bcrdr2 #1
bcrdr2 #2
cBcRdR1 #1
cBcRdR1 #2
Colony area (cm²)
0
10
20
30
40
a" a" a" a" a" a" a"
a' a' a' a' a' a' a'
ab ab ab ab ab b a
3
4
5
Days
B. cinerea WT
bcrdr1 #2
bcrdr1 #4
bcrdr2 #1
bcrdr2 #2
cBcRdR1 #1
cBcRdR1 #2
