# Supplementary material for: A fungal RNA-dependent RNA polymerase is a novel player in plant infection and cross-kingdom RNA interference
Source: PLoS Pathog. 2023 Dec 20;19(12):e1011885. doi: 10.1371/journal.ppat.1011885 (PMC10766185; doi:10.1371/journal.ppat.1011885)
Supplement: S18 Fig — Pictures were taken at 38 days after growing in short-day condition. The scale bar represents 1 cm. (PDF) [file ppat.1011885.s018.pdf]

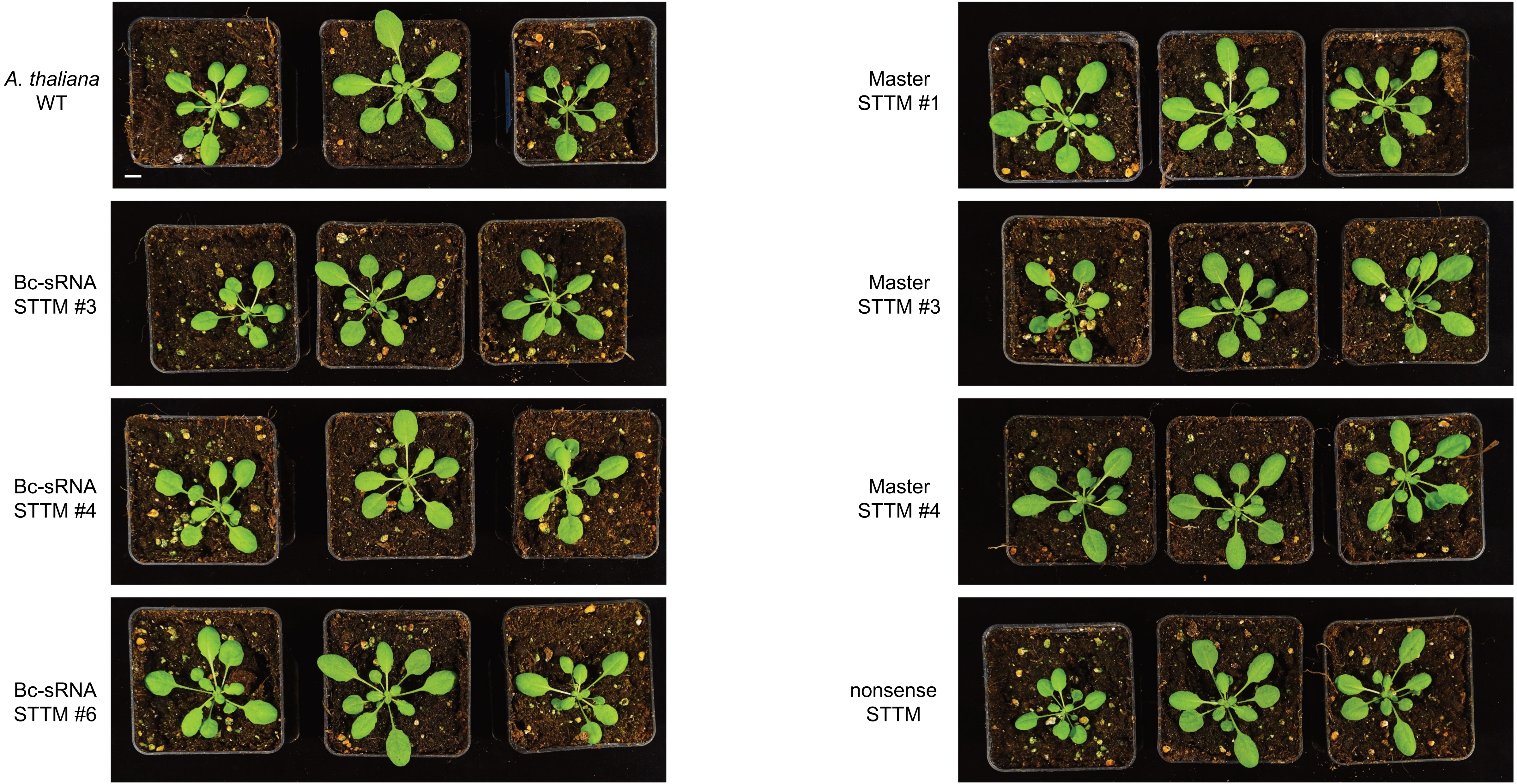

A. thaliana
WT
Bc-sRNA
STTM #3
Bc-sRNA
STTM #4
Bc-sRNA
STTM #6
Master
STTM #1
Master
STTM #3
Master
STTM #4
nonsense
STTM
