# Supplementary material for: A fungal RNA-dependent RNA polymerase is a novel player in plant infection and cross-kingdom RNA interference
Source: PLoS Pathog. 2023 Dec 20;19(12):e1011885. doi: 10.1371/journal.ppat.1011885 (PMC10766185; doi:10.1371/journal.ppat.1011885)
Supplement: Attachment — Submitted filename: Response to the reviewers.pdf [file ppat.1011885.s025.pdf]

Dear editors, dear reviewers

Thank you again for your valuable comments that helped us to improve our manuscript. Please, find below our response to all the reviewers` comments and suggestions. We have now uploaded a new version of our manuscript including minor revisions marked with yellow-track-change records.

We hope by this upgraded manuscript to satisfy you and the reviewers.

Best wishes in the name of all the authors.

Arne Weiberg

Reviewer #1: In Figure R1 the authors show that BcGypsy1 and 3 siRNAs do not result from phased biogenesis unlike tasiRNA of plant. The maximum RPM for 22-nt BcGypsy3 is just over 30.000, but in figure sup3B rep1 goes up to 120.000. how come?

Authors` response: We thank the reviewer for pointing this out. This difference in scale is only due to the units presented. Figure S7B shows the scale in abundance (number of reads). This library has 4,663,399 reads aligned to the genome and 143,392 (22-nt) reads aligning to this locus, calculating the RPM to be 30748.39. This is quite similar to what is shown in Figure R1, but any further discrepancies are explained as this is the averaging of both replicates, while S7B has them separated. Figure S7B has now been modified to show these values in RPM, which matches Figure R1 and Figure S7A.

Reviewer #1: In Figure R1, where are the atTAS1A reads sampled from? Is the data borrowed from another paper or from the authors own datasets? If so, this cannot be from axenic culture as in the data included in this study.

Authors` response: The additional library for use in Figure R1 came from Arabidopsis thaliana stem samples (PRJNA314979) and was chosen as a positive track for phasing. Phase analysis was performed by aligning reads to cDNA and genomes using ShortStack, while looking for signs of phasing in fourier transformations, size profiles, and coverage tracks of alignments. This figure shows cDNA alignment (Araport11) of AtTAS1a (AT2G27400) and genomic alignments (ASM14353v4) for the Gypsies.

Reviewer #1: In Figure S7A, it appears that most siRNA from BcGypsy3 are coming from the negative (-) strand? This is mostly consistent with Porquier et al. 2021, where most siRNA are also produced from the - strand, in axenic and infected samples. However, a fair number of reads are also coming from the + strand in that previous study. Can the authors explain the discrepancy with the current study? I also noticed a significant difference in 5' bias in this study with Porquier et al. 2021 (compare figure 2C here with figure 2D in previous paper). The bias is much less pronounced here. Could this reflect a difference in library preparation between the two studies? A difference in loading and stability could influence apparent abundance of siRNA and if a given strand is fed into AGO-complexes, raise the abundance of siRNA coming from this strand over the other and could still be compatible with direct dsRNA

production from BcRDR1. In any case, this surprising result should be discussed at the end of the study and deserves its own paragraph.

Authors` response: To our opinion, the observation of the read stranding is not so dissimilar. In both cases, there is a clear bias towards negative strand read mapping. The moderate difference in plus/minus ratio could be due to distinct sequencing depth of the two studies (Porquier et al. at 28 M reads versus Cheng et al. at 4.9 M) or due to technical reasons, as already speculated by the reviewer.

In terms of the 5′ bias, the Figure 2C of this work represents all Bc-sRNAs regardless of mapping location, whereas Figure 2D in the previous study of Porquier et al. represents 5` bias only of LTR retrotransposon-mapping Bc-sRNAs. For your consideration, please find a new plot of 5` terminal nucleotide distribution for LTR retrotransposon-derived Bc-sRNAs representing our current dataset in the Figure R2 at the end of this response letter.

Reviewer #1: In supS7D: upregulation only in WT?

Authors` response: We have now measured mRNA levels of BcGypsy1 and BcGypsy3 comparing axenic culture versus infected tissue in the two *bcrdr1* ko strains #2 and #4 by qRT-PCR, and the results are presented in the new Figure S7E. Both retrotransposons are transcribed in all conditions; however, we could only detect a significant upregulation during tissue infection in the *B. cinerea* WT strain.

Reviewer #1: Figure S7A: orientation of the transcription should be indicated with an arrow as well as the different features on the gypsy element.

Authors` response: We now have added this information into the Figure S7A, as requested.

Reviewer #1: Figure 1A, 1B and 1C should be called in the text appropriately.

Authors` response: We have now referenced the Figure 1A, 1B and 1C in the manuscript text.

Reviewer #1: Line 125-126 and 129: Why past tense?

Authors` response: We have changed the tense in the manuscript.

Reviewer #1: Line 135-136: In total, 1.3 – 4.9 million reads were mapped to the B. cinerea reference genome either unique or multiple times, as to the same read mapped to multiple chromosomal regions. Unclear sentence

Authors` response: We added the information that “1.3 – 4.9 million reads” corresponds to the range of read depth among the four sequenced libraries.

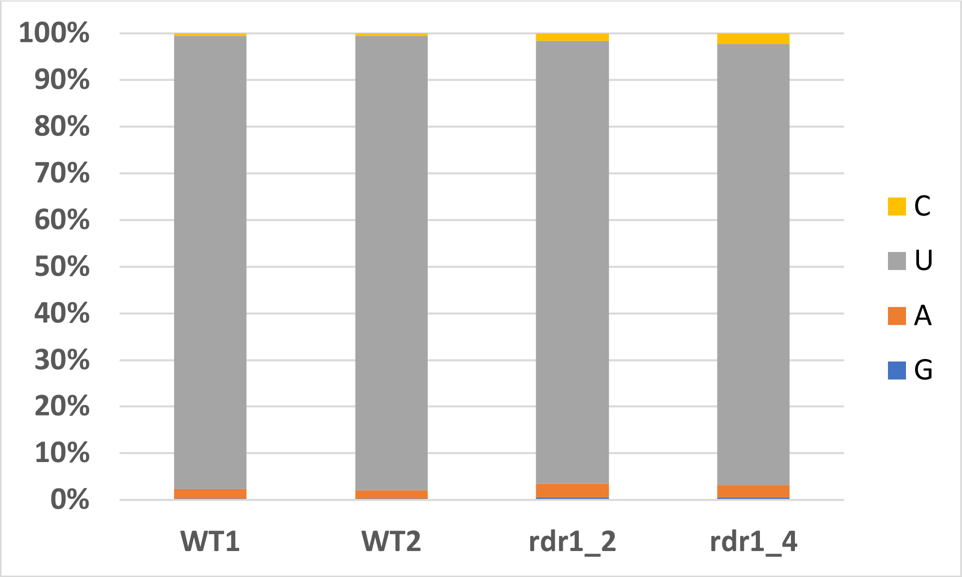

Figure R2: 5` first nucleotide base of Bc-sRNAs mapping to RTs.
