# Supplementary material for: A fungal RNA-dependent RNA polymerase is a novel player in plant infection and cross-kingdom RNA interference
Source: PLoS Pathog. 2023 Dec 20;19(12):e1011885. doi: 10.1371/journal.ppat.1011885 (PMC10766185; doi:10.1371/journal.ppat.1011885)
Supplement: Attachment — Submitted filename: Cheng et al review feedback_final.docx [file ppat.1011885.s023.docx]

The paper by Cheng et al entitled ‘A fungal RNA-dependent RNA polymerase is a novel player in plant infection and cross-kingdom RNA interference’ addresses an important and emerging question in the field that aims to understand the underlying mechanism(s) by which fungal sRNAs are generated that function in ckRNAi. It focuses on the model *B. cinerea* – Arabidopsis/tomato pathosystem and builds on earlier findings showing *Bc*-sRNAs are transported into the plant where they hijack the host RNAi machinery to suppress possible defence-related targets. In this paper by Cheng et al the authors focus on the role of BcRDR1 in this ckRNAi. Based on reduced virulence of *rdr1* ko mutants in Arabidopsis and tomato and attenuated ckRNAi in ko lines the authors propose that RDR1 is a pathogenicity factor. Although this paper makes exciting findings that link RDRs with ckRNAi there are issues with the manuscript that requires revision before it can be published and as such our initial recommendation would be to invite resubmission with major revision.

**Major comments:**

1: Through “genome survey” the authors identify 3 BcRDRs in *Bc* and show in Suppl Fig 2 that all three genes are transcribed when the fungus is grown axenically. They then go on to generate a mutant in only one of the isoforms, *BcRDR1 (*homolog of *NcSAD1* involved in MSUD*).* Why not RDR2 or RDR3 as well? I understand that RDR3 does not have homology to any well-characterised RDRs in fungi so exploring BcRDR3 role might be a bit of a dead end (although its downregulation during infection might be very interesting), but given that RDR2 is an orthologue of NcQDE1, might it not be worth exploring too?

-  	Given the jump from the above paragraph Line 88-102, where all three BcRDRs are discussed, to just focussing on RDR1 in future experiments, I feel that some justification for the sole focus on RDR1 is required especially since all three RDRs contain the conserved motif needed for RNA polymerase function.

2. Using *bcrdr1* knockout (ko) mutants the authors show mutants have reduced lesion size and fungal biomass on Arabidopsis and tomato detached leaves while axenically grown ko mutants appear comparable to WT (Fig 1A-D and Fig S3). In Lines 107-108 based on these results the authors conclude that BcRDR1 is a pathogenicity factor required to generate sRNAs needed for ckRNAi. Surely a reduction in the ability of Bc to cause disease, ie the virulence of ko *Bc* strains would also produce a reduction in lesion size during infection which may be unrelated to ckRNAi? For example, in Bc ko lines a compromise in necrosis inducing ability (e.g. failure to induce CWDE in ko lines) would also result in reduced lesion size and consequently a reduction in the production of Bc-sRNAs so results presented could be unrelated to ckRNAi. A more in-depth characterisation of fungal virulence (rather than just measuring axenic mycelial growth and BcTUB expression in infected tissue) should be provided.

3. In Fig S2 the authors show expression of all 3 RDRs are suppressed during infection. However, in an earlier publication (Weiberg et al 2013) the authors show candidate Bc-siRNAs from this study are significantly upregulated during infection. Therefore, it seems counterintuitive/unlikely that RDR1 is required for the generation of Bc-sRNAs involved in ckRNAi during infection. Please could the authors clarify/comment on this?

4. Attenuated expression of Bc-sRNAs is shown in ko lines grown axenically. However, based on Weiberg et al 2013 showing Bc-sRNAs upregulated during infection to confirm if Bc-sRNAs are RDR1-dependent could the authors please confirm that Bc-sRNA expression is compromised in *bcrdr1* ko lines in infected tissue (normalised to fungal biomass)?

5. In line 124-126 the authors show that “accumulation of RT-derived Bc-sRNAs in *bcrdr1* ko mutants resulted in increased mRNA levels of BcGypsy3, (Fig S4), suggesting a role of BcRDR1 also in post-transcriptional silencing of RTs.” FigS4 shows both BcGypsy1 and 3 are upregulated in ko line 4.

- Could the authors show RDR1-dependent sRNAs that would target BcGypsy1/3?

- What would the consequence be of upregulated BcGypsy1/3 on the fungus especially ito its virulence?

4. The authors generate a ‘switch-on’ reporter to monitor ckRNAi and focus on two sRNA targets for Bc-sRNAs (siRNA3.1 and 3.2). Could the authors explain why they chose these two siRNAs (which are generated from the same locus) over siRNA5 and siRNA20?

- As a matter of interest, could the authors clarify why both target sites were included in the reporter rather than testing Bc-sRNA target sites individually (perhaps in duplicate) in the reporter? With both included it remains unclear if only one or both are targeted by Bc-sRNAs. Please clarify?

- To demonstrate specificity for target sequences scrambled control target sequence should be included or a control Bc sRNA target that is not modulated through ckRNAi. This data should be included.

- In Line 152-153 “*Csy4 constantly suppresses expression of the GFP, unless GFP expression is activated when Bc-sRNAs silence Csy4*.” However, Fig 4D shows GFP expression in WT during infection comparable to that of non-infected plants. Indeed, only one datapoint in WT shows slightly elevated GFP transcript levels. As such this data is not convincing as excluding the single datapoint would show no difference in GFP expression in the presence or absence of Bc.

- Similarly, the WB in Fig 4E also shows presence of GFP in reporter lines in the absence of the fungus. This suggests a leakiness in the reporter system which could be due to Bc-sRNA target sites being targeted by endogenous plant sRNAs. Could this be due to endogenous host sRNAs with potential to target the same Bc-sRNA3.1/3.2 targets and that could switch on the reporter in colonised tissue?

- Also, could reduced levels of GFP in ko lines be due to reduced levels of endogenous host sRNAs in response to lower fungal biomass? Is 10x more conidiospores mentioned in Line 172 sufficiently high especially since BcTUB is still shown to be low?

- Perhaps reporter leakiness is due to promoter (proEF) choice?

- Fig 7S shows ko line 4 also being more ‘leaky’ than line 2; please could the authors comment on this?

The authors should address these comments and include experiments/controls to validate the reporter; since with GFP ON in the absence of fungal infection, the role of RDR1 in ckRNAi remains inconclusive.

5. Figure 4B shows a time series of GFP expression in Bc infected WT reporter lines compared to the *bcrdr1* line 2 and in Fig 4C GFP intensity is quantified. Could the authors please include a control that lacks GFP to show that the fluorescence observed is not due to autofluorescence from colonised tissue. Similarly, could the authors stain fungal hyphae with WGA-Alexafluor or Trypan Blue to confirm if GFP reporter activation coincide with the fungal colonisation – especially at the infection front as shown in Fig S7B.

**Line-specific comments**

Lines 38-39: “The cellular-type RDRs are conserved in several eukaryote kingdoms, including plants, nematodes, and fungi”

-  	If using the term “kingdoms”, nematodes should be listed under animals – if wanting to make the point that RDRs are found in some animals like nematodes, but not in e.g. mammals, this should be reworded

Line 88: SPAG- “we” not “were”

Line 89: “Through a genome survey”

-  	What does genome survey mean in this context? Genome survey often refers to genomic structure which I assume the authors are not referring to, thus how were the three RDR-encoding genes identified - by homology to characterised RDRs (e.g. SAD-1/QDE-1), gene annotation searching, protein domain analysis? This specific approach used should be clarified here, and should also be described in Materials and Methods.

-  	On a related note, I would advise the authors to include greater detail with respect to any *in silico* analyses (both this and phylogenetic analysis) in the supplementary materials. While gene loci identifiers are provided throughout, a table describing the source of the sequence data used (e.g. NCBI/Phytozome/ensemblPlants) and the date this resource was accessed will greatly improve replicatibility of the study - as would the transcript IDs used in phylogenetic analysis.

Lines 98-99: “however, there was no significant up-regulation of any of the BcRDRs measured during infection (Figure S2A).”

-  	In fact, there appears to be significant downregulation of RDR1 at 2dpi, and RDR3 from 1dpi onwards- please could the authors comment on/ discuss the implications thereof?

Lines 104-105: “lower fungal biomass, as estimated by quantification of *B. cinerea Tubulin* mRNAs levels”

-  	The authors later state axenic growth is not impaired in the mutants but it would be reassuring to see that *BcTubulin* expression is also not affected in the knockout mutants relative to wild type strains to provide additional direct evidence for the authors’ conclusion. Indeed, this would be particularly useful to show in relation to Figure S7E which shows reduced *BcTubulin* expression in mutants even with 10x greater conidia concentrations used for inoculation.

Lines 107-108: “Based on these results, we assume that BcRDR1 is a pathogenicity factor in B. cinerea”

-  	Careful of wording here- “assume” implies a judgement without evidence- better to say “we believe”, or “BcRDR1 is likely a pathogenicity factor in B.cinerea”

Line 113: “B. cinerea Bc-sRNA biogenesis”

-  	Redundant- either “B.cinerea sRNA biogenesis” or “Bc-sRNA biogenesis”

Line 130: SPAG- “**Vacuolar**” not “valuolar”

Line 133: “and the *A. thaliana* host genes AtMPK1 (AT1G10210), AtMPK2”

-  	All other genes are given full names here, these should be too

Line 134: “**Peroxiredoxin**” not “Peroxredoxin”

Lines 141-142: “S. lycopersicum Sl Proteinase inhibitor (PI)-I and SlPI-II or A. thaliana AtPlant Defensin (PDF)1.2 immunity genes”

-  	Redundancy- can remove the “Sl” and “At”

Paragraph 145- 176

-  	Is it correct to claim that this is a “novel” reporter system for ckRNAi- to my knowledge this has been done before in the Dunker et al. 2020 paper- I understand that the GUS has been switched out for GFP in this case but does this really add this much novelty, as it is still operating on the same principles

-  	To be accurate this should be rephrased to better reflect the adaptation of this ckRNAi reporter and the Dunker 2020 paper should be cited as the first instance of using this Csy4-based ckRNAi reporter– also, why was the decision made to switch out GUS for GFP? This does not necessarily need to be explained in the main text but would be interesting to know.

-	Lines 174-176 refer to observations that strongest GFP activity was identified at the infection front after inoculation with WT *B. cinerea* but, while interesting, it is not immediately apparent how this relates to the overarching results that *bcrdr1* mutants are compromised in ckRNAi - was this observation not found upon inoculation with the mutant strains?

-	How did the authors discriminate between increased GFP expression in infected material and increased autofluorescence due to higher fungal infection compared to ko lines. Please could the authors provide controls to discriminate between GFP signal and autofluorescence.

Lines 191-193: “Bc-sRNAs STTM plants exhibited reduced lesion sizes induced by B. cinerea, compared to *A. thaliana* WT or a transgenic *A. thaliana* line expressing a non-sense RNA-STTM”

-  	I am assuming that the authors mean a “nonsense” STTM- i.e. one with a scrambled sequence that will not lock the sRNAs- this is made a bit unclear with the hyphenation of “nonsense” to “non-sense” which to me implies something related to sense and antisense strands- I think this should be changed to make the meaning clearer (again in line 195 and in figure 5B, 5C, S8)

Lines 210-211: “*A. thaliana* master STTM plants grew and developed normally and did not show any pleiotropic defect.” Please could the author show the data?

Lines 215-216: “Reduced infection was not due to constantly enhanced plant immunity”

-  	The authors’ conclusion is drawn from evidence that *Pseudomonas syringae* infection is not affected in the master STTM plants. While this is useful evidence, this is assuming fungal, oomycete and bacterial pathogens all elicit the same immune response. Better evidence for this claim would be to show that the expression of plant immunity-associated genes (other than those targeted by the pathogens) in the master STTM plants is not affected upon inoculation with the assayed pathogens.

Lines 217-218: “did not result in fewer reduced bacterial colonies” -> either fewer or reduced, not both

Lines 230-232: The authors mention the *bcdcl1dcl2* ko in the *ku70* background that showed no evidence for ckRNAi – what point is the authors making here?

Lines 268-270: “It will be interesting to investigate whether microbial RDRs play a broader role in diverse plant-microbe interactions and in cross-kingdom RNA communication.”

-  	As the targeting of BcDCLs using SIGS has already been mentioned in this paragraph, it might be nice to further extend this conclusion to suggest how this research could be extended to have some practical applications i.e. the identification of a new fungal pathogenicity factor (BcRDR1) that could similarly be targeted using SIGS in disease control

Line 325: “using a binocular.” -> “using a binocular **microscope**.”

Line 378: “by default multiple alignment algorithms.”

-  	While the authors have included the software and version number used in this analysis, this software is proprietary and all readers may not have access to it. It would be nice to at least include what these default settings are, even if just as “... algorithms (gap open cost: X; gap extension cost: Y).” to enable replication using alternative software.

Line 505: “The scale bars in A) and C) represent 500 μm”

-  	Should this be “A) and B)”?

Figure S1:

-  	This figure would benefit from an explanation of the ‘Max’ and ‘Min’ colour spectrum used within (whether in the caption or by adjusting these labels). Does this refer to residue conservation or another measure of alignment/homology?

Figures S6-S7

-  	The brightfield images should be included as they were taken (i.e. before merging with GFP) such that the figures show BF, GFP, merged.
