# Supplementary material for: A fungal RNA-dependent RNA polymerase is a novel player in plant infection and cross-kingdom RNA interference
Source: PLoS Pathog. 2023 Dec 20;19(12):e1011885. doi: 10.1371/journal.ppat.1011885 (PMC10766185; doi:10.1371/journal.ppat.1011885)
Supplement: Attachment — Submitted filename: response letter to editor and reviewers.docx [file ppat.1011885.s024.docx]

**Response letter:**

**Dear Editors, dear reviewers,**

We would like to thank you for reviewing our manuscript entitled “A fungal RNA-dependent RNA polymerase is a novel player in plant infection and cross-kingdom RNA interference“, and appreciate all the helpful comments that has improved our work. In the following letter, we have responded to all the given comments, and provide a summary of our additional works requested by the reviewers. We provide a revised version of our manuscript with tracked changes. By this, we hope that could clarify all the open points and to satisfy all the issues and questions.

Best wishes in the name of all the co-authors,

Arne Weiberg

**Editors´ comments:**

The reviewers appreciated the nice study on an intriguing aspect of plant-pathogen interactions. While they generally appreciated the novel findings presented in the study, they also raised a number of important points.

The reviewers appreciate the elegant use of the “switch-on” GFP reporter system. However, they also ask for additional validation and controls for this part of the study. In the revised version of the manuscript the authors should make sure that these important results are convincingly generated and presented.

The authors should justify the focus on RDR1. As pointed out in the reviews, RDR2 or RDR3 could likewise have a functional relevance in virulence. Hereby, the authors should also interpret their data with respect to previously published results from Weiberg et al, 2013 and discuss the role of RDR1 in the generation of sRNAs involved in ckRNAi.

The authors conclude that reduced virulence of the RDR1 knock-out mutant implies a functional relevance of cross-kingdom RNAi in virulence. As correctly pointed out by the reviewers, the reduced virulence could potentially be caused by other functions related to small RNAs. The authors should clarify their argumentation and more precisely address the role of RDR1. The study does not include a complementation test of the Botrytis RDR1 mutant. As pointed out by a reviewer, complementation of the rdr1 mutant should be done to validate the functional relevance in ck-RNAi.

One concern also relates the to the small RNA sequencing. From the figures and text it is unclear if the small RNA sequence data is phased or not. This has importance for the interpretation of the data and should therefore be clarified.In a revised manuscript, the authors should respond to all comments raised by the three reviewers.

**Authors’ response to the editors:** We thank the editors for handling our manuscript and to extract the relevant points how to improve our work. In our revised manuscript, we now have included data for the following requested experiments:

I) Phenotype and molecular analysis of two independent cBcRDR1 genetic complementation strains.
II) Additional control experiments regarding the GFP switch-on cross-kingdom RNAi reporter. These include a) molecular data presenting GFP activation during the time course series by qRT-PCR and Western blot analysis, b) a GFP time course series with water-treated reporter plants, c) a time course series with non-GFP expressing WT A. thaliana plants infected by B. cinerea, d) infection assays of the GFP reporter plants with the oomycete pathogen *Hyaloperonospora arabidopsidis*. With these additional experiments, we rule out that measured GFP activation was not due to auto-fluorescence nor to unspecific, stress-related infection effect, but truly induced by cross-kingdom RNAi.
III) Analysis of phasing small RNAs at the *BcGypsy1* and *BcGypsy3* loci.
IV) Comparative expression analysis of five virulence factors in the *B. cinerea* WT and *bcrdr1* ko mutant strains.

Further on, we now give additional explanation and justification why we chose the BcRDR1 in this study. With these new analysis and data in our revised work, we hope to convince you and the reviewers on the functional role of BcRDR1 in *B. cinerea* pathogenicity and cross-kingdom RNAi.

**Reviewer's comments**: **Major Issues and Key Experiments Required for Acceptance**

**Reviewer #1**: The authors detect three putative BcRdR proteins via phylogeny, but focus on RdR1 without providing an explanation. The authors should provide an explanation for focusing on that gene rather than the others. I also think knock out of the two other RdR genes could yield insight into possible redundancy between RdRs in ckRNAi and small RNA biogenesis or lead to a developmental phenotype in the fugus, like it is the case for the double DCL mutant.

**Authors’ response:** The main reason why we focus on the BcRDR1 in this study is its role in pathogenicity and cross-kingdom RNAi. We agree that investigating the role of BcRDR2 and BcRDR3 in *Botrytis* is worth doing. That is why we now attempted to generate *bcrdr2* and *bcrdr3* ko mutants, as well. We now present new data of two isolated *bcrdr2* ko mutants (updated Figure 1). The *bcrdr2* ko mutants did not exhibit any altered infection phenotype. In the case of BcRDR3, we only reached to heterokaryotic fungal isolates. One reason for this outcome could be that *bcrdr3* ko could have a lethal phenotype.

**Reviewer #1**: In order to demonstrate the role of BcRdR1, the mutant lines should be complemented by a WT transgenic copy of the gene, which should restore small RNA production. Additionally, a similar construct with a catalytically dead version of the enzyme should be generated, in order to test if production of dsRNA from the Gypsy locus is a prerequisite to BcsRNA production.

**Authors’ response:** We appreciate the reviewer´s comment. As suggested, we have now generated two independent BcRDR1 complementation strains, cBcRDR1 #1 and #2, in the *bcrdr1* ko mutant background. cBcRDR1 complementation strains were confirmed by genotype PCR and RT-PCR (updated Figure S3C). Infection of tomato and Arabidopsis with the cBcRDR1 strains induced lesion size and revealed fungal DNA content similar to the *B. cinerea* WT infection (updated Figure 1). Moreover, cBcRDR1 strains produced Bc-sRNAs during tomato infection (updated Figure 3B). These new data further support the functional role of BcRDR1 in fungal pathogenicity and Bc-sRNA production.

**Reviewer #1**: Regarding the small RNA population affected in rdr1 mutant: are reads coming from both strands, as would be expected from sRNA produced from a dsRNA precursor? Since the population of sRNA appears non random from the figure S4A (although it is quite hard to see), are the small RNA phased? Phasing could hint at their biogenesis mode (resulting from a single fungal RISC cleavage/stalled ribosome – downstream recruitment of RdR1?) as seen for plant tasiRNA and LTR retrotransposons.

**Authors’ response:** We thank the reviewer for this insightful thoughts. We performed phasing analysis at *BcGypsy1* and *BcGypsy3* loci following the PhaseTank tool strategy (Guo et al., 2015). We used the *Arabidopsis* *AtTAS1a* locus as a known reference for phasiRNA production in plants. Comparing these loci, there was no hint for phasing small RNAs in *B. cinerea* (Figure R1). However, we would like to emphasize that phasing analysis in *B. cinerea* is greatly limited by unknowns. We don’t have any sense of the phasing small RNA sizing in *Botrytis*. Also, we don’t know what triggers could cause phasing small RNA production in *Botrytis*. We are greatly limited in our ability to identify small RNA targeting in *Botrytis*, if this is required as a trigger. For this reason, it was not attempted. In addition, most *BcGypsy1* and *BcGypsy3*-derived Bc-sRNAs are strand-specific, while phasing small RNAs in plants typically appear at both stranded.

**Reviewer #1**: In the rdr1 mutant, are the remaining sRNA on gypsy elements still mainly 22nt or 24nt as suggested by figure 2b? This should be made clear in figure S4A (color code legend?).
**Authors’ response:** We now show strand-specific coverage plots of BcsRNAs at the *BcGypsy*s loci and include respective BcsRNA size profiles in the updated Figure S7.

**Reviewer #1**: Figure 3B is overcropped: there appears to be additional signal under and above the central lane. Please show the uncropped gel. In general, raw data should be made available for all figures.

**Authors’ response:** We now provide uncropped gel images in the new Figure S8. We further performed and present stem-loop RT-PCR data of Bc-sRNAs with samples collected from *B. cinerea*-infected tomato (updated Figure 3B). We now give all raw data of our qPCR and qRT-PCR experiments in the new Table S3.

**Reviewer #1**: Figure 4B is lacking a negative control: t0 of treatment and mock inoculated leaves.

**Authors’ response:** We have now included a time series of water-treated GFP reporter plants in the updated Figure 4 to assess auto-activation of the GFP reporter. Non-GFP expressing WT plants infected with *B. cinerea* were also tested (new Figure S11 and Figure S12) to assess auto-fluorescence.

**Reviewer #1**: Figure 4C is the quantification from a single leaf? For a robust conclusion, intensity over several treated leaves should be averaged, and deviation displayed.

**Authors’ response:** We now provide a second independent time course experiment of *B. cinerea*-infected GFP reporter plants (new Figure S10). Each data point represents the GFP signal intensity of the same leaf measured at different time points of infection. Each raw signal intensity at a given time point (I_t_) was normalized by subtraction of the initial intensity (I_0_). We moreover provide more fluorescence microscopic images coming from three independent inoculation experiments with detached leaves of adult GFP reporter plants (Figure S12).

**Reviewer #2:** Strengths and Weaknesses: In this paper, the authors investigated the role of RDR1 (one of B. cinerea's RNA-dependent RNA polymerases) in pathogenicity and, more importantly, in cross-kingdom RNAi. Their findings may help researchers understand the mechanism of cross-kingdom RNAi. They developed a switch-on GFP reporter, which is a novel technique that can be used to study cross-kingdom RNAi in different species. This technique can also be employed to investigate the role of RNA silencing complexes in both hosts and pathogens, thereby identifying the molecules responsible for RNAi—a significant question in the field of host-microbe interactions. However, the manuscript needs major improvement, which can be done by additional experiments to confirm their findings. Specifically, to highlight their novel method, the switch-on GFP reporter, it is important to validate their observations using additional methods mentioned below. In the Material and Methods section, the methodology of the experiments is unclear. The authors need to: 1- Clarify the method used to perform inoculation for all infection assays. The authors must provide clear details on how they inoculated the plants for each experiment, including the gene expression assay and switch-on GFP reporter analysis.

**Authors’ response:** We now provide more details on the methods of plant inoculation, gene expression analysis and examination procedures of the GFP switch-on reporter plants in our materials and methods chapter.

**Reviewer #2:** Specify the reference genes used for mRNA expression or small RNA expression assays. Additionally, explain how they dealt with RNA contamination from either the plant or pathogen while investigating mRNA/small RNA expression. In Figure 1C, they have briefly mentioned the reference gene for measuring biomass, but the authors need to provide detailed information in the Materials and Methods section.

**Authors’ response:** We have now added the information on the reference genes used for mRNA and small RNA expression analysis given in the respective figure legend, the material and methods section and in the updated Table S1.

**Reviewer #2:** Validate some of the microscopy data using other approaches, such as western blotting, especially for the switch-on reporter, which is one of the main techniques used in the experimental procedures and resulted in the key finding.

**Authors’ response:** We thank the reviewer for this suggestion. We now include qRT-PCR data as well as Western blot analysis of GFP expression. These data further support the findings of the microscopy study and are now included in the updated Figure 4.

**Reviewer #2:** Address the compatibility issues of some results presented in different figures/panels. The authors should either remove conflicting data or provide explanations in the text.

**Authors’ response:** We now added information to explain and clarify these issues in our revised manuscript text and in the figure panels and legends.

**Reviewer #2:** Improve the figure legends by including more detail. Authors can add a short explanation of their methodology in the figure legends, particularly for the infection assay, to clarify how the inoculation was performed in each figure or for gene expression assay.

**Authors’ response:** We have now added more information regarding inoculation methods and gene expression analysis in the respective figure legends and the materials and methods section.

**Reviewer #2:** Simplify or rewrite some paragraphs that are complicated to understand, making them easier to follow for readers.

**Authors’ response:** We improved our manuscript text by reducing complexity and simplified the wordings.

**Major comments and suggestions based on text and figures:**

**Reviewer #2:** Line 124 to 126: The authors reported that the mRNA level of BcGypsy3 increases in the Bcrdr1 mutant, suggesting that BcRDR1 may play a role in post-transcriptional silencing of retrotransposons (RTs). However, the authors' confidence in this statement is not explicitly mentioned. It is possible that the accumulation of BcGypsy3 mRNA in the Bcrdr1 mutant is a result of compromised sRNA production in Bcdrd1. In the wild-type B. cinerea, RDR1 is involved in generating dsRNA molecules from single-stranded BcGypsy3 mRNA, which are subsequently targeted by DCLs (Dicer-like proteins), leading to the production of small RNAs (sRNAs). However, in the bcrdr1 mutant, the BcGypsy3 mRNAs are not affected by this process, potentially resulting in the accumulation of BcGypsy3 mRNA. Further investigations are needed to ascertain the precise role of BcRDR1 in the post-transcriptional silencing of RTs and its impact on sRNA production in B. cinerea.

**Authors’ response:** As the reviewer pointed out correctly, we are very careful to drive any strong conclusion on a potential regulatory mechanism of Gypsy-type LTR retrotransposons by the BcRDR1 at this stage. The reason is that on the one hand, *bcrdr1* ko mutants indicated higher mRNA level of *BcGypsy3*, and *BcGypsy1* in one of the *bcrdr1* ko mutants (Figure S7C). This could indeed mean, as suggested by this reviewer, that BcRDR1 is required to generate dsRNAs from single-stranded *BcGypsy* mRNA precursors, followed by processing into siRNAs by BcDCLs and *in cis* silencing of *BcGypsy* mRNAs. On the other hand, BcsRNAs mapping at the *BcGypsy* loci are mostly strand-specific (updated Figure S7A) that speaks rather against accumulation of dsRNA precursors. Nevertheless, *BcGypsy*-related Bc-sRNA biogenesis is clearly dependent on BcRDR1 (updated Figure S7A-B). We agree with the reviewer that ascertain the precise role of BcRDR1 in retrotransposon silencing would require additional investigation. We think that this aspect, however, lies beyond the scope of this work, which is to present data that prove the functional role of BcRDR1 in fungal pathogenicity and cross-kingdom RNAi.

**Reviewer #2:** Line 139: I couldn't find Figure 3D mentioned in line 139. Figure 3 only has three panel: A, B, and C. Please clarify which panel belong to 3D.

**Authors’ response:** We apologize for this mistake and have now corrected it.

**Reviewer #2:** line 139 to 144 (related to both figure 3C and S5A,B) the authors stated that they measured successful infection by analyzing the expression pattern of S. lycopersicum SlPI-I and SlPI-II or A. thaliana AtPDF1.2 immunity genes. If the infection is successful and B. cinerea possesses effective pathogenicity, it is expected to observe up-regulation of AtPDF1.2 in arabidopsis. However, in Figure 3C and Figure S5A-B, Arabidopsis infected by bcrdr1 is actually showing a higher expression of AtPDF1.2, which indicates that bcrdr1 and bcrdr2 mutants have a higher level of infection, does that mean higher level of pathogenicity? If yes, this finding is inconsistent with the authors' conclusion in line 139 to 144.

**Authors’ response:** According to our statistical analysis represented in Figure 3C and Figure S9B, no difference was observed in pathogen-induced *AtPDF1.2* expression levels comparing *B. cinerea* WT and *bcrdr1* ko mutants, as indicated by the letter code representing the results of a one-way ANOVA test. We think that this data is an important piece of information to present, because *AtPDF1.2* is not a direct target gene of BcRDR1-dependent BcsRNAs, in contrast to the target genes *AtPRXIIF*, *AtMPK1* and *AtWAK*. Therefore, *AtPDF1.2* was not expected to over-accumulate in the *bcrdr1* ko mutants, as this was the case for the *Arabidopsis* target genes. The induction of the *AtPDF1.2* expression provided additional validation of proper infection with all *B. cinerea* strains used in this assay. We do not intent to conclude on any quantitative correlation between *AtPDF1.2* expression level and pathogenicity strength.

**Reviewer #2:** Line 309 to 313: This paragraph is unclear. Although the paper contains numerous infection and pathogenicity assays, it is not clear how they performed the infection assay. For instance, in lines 317 to 321, it states: "For recording GFP cross-kingdom RNAi reporter time course, 3-week-old A. thaliana were cultivated on 1⁄2 MS + 1% sucrose agar plates and inoculated with 2x105 conidia/ml B. cinerea resuspended in 1% malt extract medium pre-incubated for 1 hour. The pre-incubated conidia suspension was washed twice with sterile water before 5 μl was dropped at the center of one leaf per seedling." It is unclear whether they sprayed conidia over the leaf or only used a droplet at the center. If they used a 5 μl droplet as mentioned in the text, the authors need to explain how 5 μl droplet of conidia in the center of the leaf can cause infection in the entire leaf and induce the reporter in the entire leaf, as presented in Figure 4B and supporting video 1.

**Authors’ response:** We thank the reviewer for this helpful comment. We have now clarified the two different inoculation procedures of the GFP reporter plants in the materials and methods section. We infecting leaves of whole seedlings of the GFP reporter plants growing on agar petri dishes to record infection time course series. A drop of *B. cinerea* conidiospores was placed onto the leaf followed by topping with a cover glass slip. This led conidiospores disperse over the entire leaf. We now give this information in our manuscript text and in the figure legend to let the reader understand, why the GFP reporter is activated over the entire leaf during the time series experiments.

**Reviewer #2:** Figure 4B: This is one of the key results and requires additional validation through another method, such as western blotting. To confirm the findings, it is recommended to repeat the same experiment using same timepoints and perform a western blot using anti-GFP antibodies to demonstrate that higher expression of GFP only occurs when plants are infected by WT B. cinerea. To ensure that each sample contains the same amount of protein, either Ponceau staining or silver staining can be used.

**Authors’ response:** We now have included this data in the updated Figure 4, as requested. The results are fully consistent with the microscopy images and clearly confirm activation of GFP during infection with *B. cinerea* WT, but not *bcrdr1* ko mutants. As a protein loading control in Western blots, we stained the blotting membrane with Coomassie Brilliant Blue and used the RuBisCo band as a reference.

**Reviewer #2:** Figure S2A: In this figure, the authors aimed to evaluate the expression pattern of RDR1, RDR2, and RDR3 in B. cinerea both in axenic media and during infection. Line 98 to 99: they were unable to detect any significant up-regulation during infection, but they did not provide detailed information about the experiment in method section. One of the main challenges in evaluating gene expression during infection is dealing with the ratio of plant and fungal pathogen RNAs. The main questions raised are: What is their RNA isolation method? How did they quantify plant or fungal RNAs? And what reference genes were used to normalize the amount of plant or fungal RNAs?

**Authors’ response:** We thank the reviewer for this point. We now include the requested information in our materials and methods section and give additional information in the respective figure legends.

**Reviewer #2:** Figure S6: Similar to what has been suggested for the result in Figure 4B, here I also recommend using western blotting (WB) and anti-GFP antibodies to validate your result. This can be achieved by using WT B. cinerea, bcrdr1#2, and bcdcl1/2 at two time points: 6 hours and 24 hours post-infection.

**Authors’ response:** We now provide molecular data of GFP activation during the infection course with *B. cinerea* WT and *bcrdr1* ko mutants by qRT-PCR and Western blot analysis (updated Figure 4). These data provide more evidence for GFP induction only during *B. cinerea* WT infection, but not *bcrdr1*. In our study, we used the *bcdcl1dcl2* ko as another control strain that lost Bc-sRNA production together with water-treated GFP reporter plants as well as non-GFP expressing *Arabidopsis* WT plants (Figure 4, Figure S10, Figure S11 and Figure S12) in order to confirm that GFP induction is truly due to *B. cinerea* cross-kingdom RNAi. Thus, we feel that measuring GFP expression at different time points by qRT-PCR or Western blot in samples with no GFP activation as observed by fluorescence microscopy would not provide any additional valuable information in this context.

**Reviewer #3:** 1: Through “genome survey” the authors identify 3 BcRDRs in Bc and show in Suppl Fig 2 that all three genes are transcribed when the fungus is grown axenically. They then go on to generate a mutant in only one of the isoforms, BcRDR1 (homolog of NcSAD1 involved in MSUD). Why not RDR2 or RDR3 as well? I understand that RDR3 does not have homology to any well-characterised RDRs in fungi so exploring BcRDR3 role might be a bit of a dead end (although its downregulation during infection might be very interesting), but given that RDR2 is an orthologue of NcQDE1, might it not be worth exploring too? Given the jump from the above paragraph Line 88-102, where all three BcRDRs are discussed, to just focussing on RDR1 in future experiments, I feel that some justification for the sole focus on RDR1 is required especially since all three RDRs contain the conserved motif needed for RNA polymerase function.

**Authors’ response:** The main reason why we focus on BcRDR1 in this study is the discovery of its role in pathogenicity and cross-kingdom RNAi. We agree that investigating the roles of BcRDR2 and BcRDR3 in *Botrytis* is worth doing. That is why we now attempted to generate *bcrdr2* and *bcrdr3* ko mutants, as well. We now present new data of two isolated *bcrdr2* ko mutants (updated Figure 1). The *bcrdr2* ko mutants exhibited no altered infection phenotype. In the case of BcRDR3, we only reached to heterokaryotic fungal isolates. One reason for this outcome could be that *bcrdr3* ko could have a lethal phenotype.

**Reviewer #3:** 2. Using bcrdr1 knockout (ko) mutants the authors show mutants have reduced lesion size and fungal biomass on Arabidopsis and tomato detached leaves while axenically grown ko mutants appear comparable to WT (Fig 1A-D and Fig S3). In Lines 107-108 based on these results the authors conclude that BcRDR1 is a pathogenicity factor required to generate sRNAs needed for ckRNAi. Surely a reduction in the ability of Bc to cause disease, ie the virulence of ko Bc strains would also produce a reduction in lesion size during infection which may be unrelated to ckRNAi? For example, in Bc ko lines a compromise in necrosis inducing ability (e.g. failure to induce CWDE in ko lines) would also result in reduced lesion size and consequently a reduction in the production of Bc-sRNAs so results presented could be unrelated to ckRNAi. A more in-depth characterisation of fungal virulence (rather than just measuring axenic mycelial growth and BcTUB expression in infected tissue) should be provided.

**Authors’ response:** We thank this reviewer for raising this point. We now provide a more in-depth characterization of differential expression for *Botrytis* virulence genes. We measured mRNA levels of 5 known virulence genes, the *Polygalacturonase BcPG1*, the *Necrosis-inducing peptide BcNEP1*, the secreted *Cerato-platanin Spl1*, the *Endo-1,4-beta-xylanase BcXyn11A* and the *Necrosis-inducing effector HIP1* (Leisen et al., 2022), and found no difference between *B. cinerea* WT and the two *bcrdr1* #2 and #4 ko mutant strains (new Figure S6). While we cannot rule out any change of other virulence gene expression, this result however further supports that the reduced virulence phenotype of *bcrdr1* ko mutants is likely related to the loss of cross-kingdom RNAi.

**Reviewer #3:** 3. In Fig S2 the authors show expression of all 3 RDRs are suppressed during infection. However, in an earlier publication (Weiberg et al 2013) the authors show candidate Bc-siRNAs from this study are significantly upregulated during infection. Therefore, it seems counterintuitive/unlikely that RDR1 is required for the generation of Bc-sRNAs involved in ckRNAi during infection. Please could the authors clarify/comment on this?

**Authors’ response:** In a previous study, enhanced BcGypsy3-derived BcsRNA accumulation was detected during plant infection (Porquier et al., 2021). We now compared mRNA levels of *BcGypsy1* and *BcGypsy3* between axenic growth condition and tomato infection and revealed a clear up-regulation during infection (new Figure S7D). When analyzing mRNA expression levels of the three BcRDRs by and qRT-PCR in two independent inoculation replicates of tomato infection, we did not find clear regulation of *BcRDR1* (Figure S2A). Based on these results, we think that BcRDR1 is not regulated during infection, but enhanced accumulation of Bc-sRNAs is due to the transcriptional induction of the *BcGypsy* mRNAs.

**Reviewer #3:** 4. Attenuated expression of Bc-sRNAs is shown in ko lines grown axenically. However, based on Weiberg et al 2013 showing Bc-sRNAs upregulated during infection to confirm if Bc-sRNAs are RDR1-dependent could the authors please confirm that Bc-sRNA expression is compromised in bcrdr1 ko lines in infected tissue (normalised to fungal biomass)?

**Authors’ response:** We now provide stem-loop RT-PCR data of the Bc-sRNAs during tomato infection (updated Figure 3B). No Bc-sRNA signals were obtained for the *bcrdr1* ko mutants, in contrast to *B. cinerea* WT and cBcRDR1 strains.

**Reviewer #3:** 5. In line 124-126 the authors show that “accumulation of RT-derived Bc-sRNAs in bcrdr1 ko mutants resulted in increased mRNA levels of BcGypsy3, (Fig S4), suggesting a role of BcRDR1 also in post-transcriptional silencing of RTs.” FigS4 shows both BcGypsy1 and 3 are upregulated in ko line 4. Could the authors show RDR1-dependent sRNAs that would target BcGypsy1/3? What would the consequence be of upregulated BcGypsy1/3 on the fungus especially ito its virulence?

**Authors’ response:** We are very careful to drive any strong conclusion for a potential regulatory mechanism of Gypsy-type LTR retrotransposons by the BcRDR1 at this stage. The reason is that one the one hand, *bcrdr1* ko mutants indicated higher mRNA level of *BcGypsy3*, and *BcGypsy1* for one of the *bcrdr1* ko mutants (Figure S7C). This could mean that BcRDR1 is required to generate dsRNAs from single-stranded *BcGypsy* mRNA precursors, followed by processing into Bc-sRNAs and *in cis* silencing of *BcGypsy* mRNAs. On the other hand, Bc-sRNAs mapping at the *BcGypsy* loci are mostly strand-specific (updated Figure S7A), which rather speaks against production of dsRNA precursors; nevertheless, *BcGypsy*-related Bc-sRNA biogenesis is clearly BcRDR1-dependent (updated Figure S7A-B). It is therefore hard to speculate if and what could be the consequence of up-regulated *BcGypsy* mRNA expression in the *bcrdr1* ko mutant background on fungal virulence.

**Reviewer #3:** 4. The authors generate a ‘switch-on’ reporter to monitor ckRNAi and focus on two sRNA targets for Bc-sRNAs (siRNA3.1 and 3.2). Could the authors explain why they chose these two siRNAs (which are generated from the same locus) over siRNA5 and siRNA20? As a matter of interest, could the authors clarify why both target sites were included in the reporter rather than testing Bc-sRNA target sites individually (perhaps in duplicate) in the reporter? With both included it remains unclear if only one or both are targeted by Bc-sRNAs. Please clarify?

**Authors’ response:** We chose the two BcsRNA3.1 and BcsRNA3.2, because they were shown to induce cross-kingdom RNAi in *Arabidopsis* and tomato (Weiberg et al., 2013) (Figure 3A). Choosing two different Bc-sRNAs and two different target sites increased the chance to induce silencing of the *Csy4* repressor. We agree with the reviewer that it remains unclear if individual or both Bc-sRNAs induced reporter activation. As a matter of fact, we here used the reporter assay to visualize and to monitor cross-kingdom RNAi in the infected plant tissue and not to specify the effect of individual Bc-sRNAs on cross-kingdom RNAi.

**Reviewer #3:** To demonstrate specificity for target sequences scrambled control target sequence should be included or a control Bc sRNA target that is not modulated through ckRNAi. This data should be included.

**Authors’ response:** We thank the reviewer for this suggestion. We now include additional control experiments that validate the specificity of the GFP reporter assay. First, we infected the Bc-sRNA-specific GFP reporter plants with the oomycete pathogen *Hyaloperonospora arabidopsidis* (new Figure S13). This oomycete pathogen can induce cross-kingdom RNAi by its small RNAs during *Arabidopsis* infection (Dunker et al., 2020), but it failed to activate the *Botrytis*-specific GFP reporter due to non-compatible small RNAs. Second, we infected with *B. cinerea* the reporter plant line that expressed a Cys4 construct carrying *H. arabidopsidis* small RNA-specific target sites and a GUS reporter (Dunker et al., 2020). Hence, GUS expression was activated during *H. arabidopsidis* infection, but not during *B. cinerea* infection (new Figure S14). To generate new transgenic *Arabidopsis* reporter lines was beyond the time frame of this revision. We will include reporter plant lines expressing scrambled versions of Bc-sRNA target sites in future experiments.

**Reviewer #3:** In Line 152-153 “Csy4 constantly suppresses expression of the GFP, unless GFP expression is activated when Bc-sRNAs silence Csy4.” However, Fig 4D shows GFP expression in WT during infection comparable to that of non-infected plants. Indeed, only one datapoint in WT shows slightly elevated GFP transcript levels. As such this data is not convincing as excluding the single datapoint would show no difference in GFP expression in the presence or absence of Bc. Similarly, the WB in Fig 4E also shows presence of GFP in reporter lines in the absence of the fungus. This suggests a leakiness in the reporter system which could be due to Bc-sRNA target sites being targeted by endogenous plant sRNAs. Could this be due to endogenous host sRNAs with potential to target the same Bc-sRNA3.1/3.2 targets and that could switch on the reporter in colonised tissue? Also, could reduced levels of GFP in ko lines be due to reduced levels of endogenous host sRNAs in response to lower fungal biomass? Is 10x more conidiospores mentioned in Line 172 sufficiently high especially since BcTUB is still shown to be low? Perhaps reporter leakiness is due to promoter (proEF) choice? Fig 7S shows ko line 4 also being more ‘leaky’ than line 2; please could the authors comment on this? The authors should address these comments and include experiments/controls to validate the reporter; since with GFP ON in the absence of fungal infection, the role of RDR1 in ckRNAi remains inconclusive.

**Authors’ response:** We thank the reviewer for these valuable comments. We now performed both GFP mRNA expression analysis by qRT-PCR and GFP protein accumulation at different time points by Western blotting collecting samples from leaves that were taken to inspect GFP expression by fluorescence microscopy (updated Figure 4). Both qRT-PCR and Western validated a clear, gradual increase of GFP signals in plants infected by *B. cinerea* WT but not in *bcrdr1* ko mutants. Based on these results, we conclude that *B. cinerea* WT triggered GFP activation through cross-kingdom RNAi, but not the *bcrdr1* ko mutant. Indeed, low GFP protein accumulation was detected in water-treated reporter plants at similar level to *bcrdr1*-infected plants (Figure 4E). We think that this low GFP background activity is due to *proEF1* promoter strength; nevertheless, such background signal was acceptable, because GFP activation via cross-kingdom RNAi in *B. cinerea* WT-infected reporter plants was clearly and significantly up-regulated. We would rule out that less GFP activation in the case of *bcrdr1* ko mutants is due to lower fungal biomass, since infection with 10x more conidiospores resulted in indistinguishable fungal biomass of *B. cinerea* WT and *bcrdr1* ko mutant in the infected plant samples, as estimated by genomic DNA measurement via qPCR at the time point of GFP measurement (Figure S12E). We don’t think that infection with *bcrdr1* #4 induced a stronger GFP background signal than *bcrdr1* #2, when considering all three infection series replicates represented in Figure S12.

**Reviewer #3:** 5. Figure 4B shows a time series of GFP expression in Bc infected WT reporter lines compared to the bcrdr1 line 2 and in Fig 4C GFP intensity is quantified. Could the authors please include a control that lacks GFP to show that the fluorescence observed is not due to autofluorescence from colonised tissue. Similarly, could the authors stain fungal hyphae with WGA-Alexafluor or Trypan Blue to confirm if GFP reporter activation coincide with the fungal colonisation – especially at the infection front as shown in Fig S7B.

**Authors’ response:** As suggested by this reviewer, we now include fluorescence microscopic images of *B. cinerea* WT-infected *Arabidopsis* WT leaves (Figure S11). No enhanced fluorescence signal was detected at the given time points, which ruled out that any auto-fluorescence signals disturbed the GFP fluorescence measurement in GFP reporter plants. We now also include side-by-side images of the same leaf showing GFP activation at the infection site by fluorescence microscopy and fungal mycelium by Trypan Blue staining (new Figure S16).

**Part III – Minor Issues: Editorial and Data Presentation Modifications**
Please use this section for editorial suggestions as well as relatively minor modifications of existing data that would enhance clarity.

**Reviewer #1:** Why are small RNA sequenced from an axenic culture rather than during plant infection? It seems intuitive that if BcsRNA have a pathogenic role, they would be induced during infection.

**Authors’ response:** We chose to sequence small RNAs collected from axenic culture in order to avoid massive sequence flooding of plant small RNAs.

**Reviewer #1:** Similarly, it is counterintuitive that BcRdR1 decreases during infection (figure S2A). This should be commented on by the authors.

**Authors’ response:** Our data shown in Figure S2 and Figure S7D suggest up-regulation of *BcGypsy*s rather than *BcRDR1* during tomato infection. It is likely that up-regulated *BcGypsy* mRNAs caused enhanced Bc-sRNA accumulation in infected plants.

**Reviewer #1:** definition of “multiple mapping reads” is unclear: do the authors mean that they allowed mapping of single reads to multiple loci as an approximation of sRNA made from repeat elements?

**Authors’ response:** Yes, correct. We have clarified this definition in the manuscript text.

**Reviewer #1:** For the switch-on reporter, why were sites for siR3.2 and siR3.1 specifically picked for this assay? And for STTM assay, why siR3.2 and siR5? The choice of those over other described sRNA target sites is never made clear.

**Authors’ response:** The GFP reporter is a tool to show activation of cross-kingdom RNAi in the infected plant tissue. The choice of the Bc-sRNA3.1 and Bc-sRNA3.2 for the GFP reporter was because both small RNAs are largely overlapping and thus might cross-react with the bait target sites. The STTM approach is a method of choice to analyze the functional role of Bc-sRNAs for infection. The more diverse Bc-sRNAs can be trapped, the stronger the expected effect on infection.

**Reviewer #1:** There is a discrepancy between microscopy images presented in figure 4B and figure S6 and S7. The former shows accumulation of GFP in the midrib and veins, which is still visible in presence or bcrdr1 ko, while the former doesn’t show veins at al. This and the point above make it impossible to know if the leftover GFP signal is the result of incomplete Csy4 activity/insufficient expression, or of residual BdsiRNA in bdrdr1.

**Authors’ response:** This discrepancy might be explained by the fact that Figure 4 displays seedling leaves, while the Figure S12 (former Figure S6) represents adult rosette leaves that has been imaged with different microscopy set-up and magnifications. We now include a water-treated control (updated Figure 4, Figure S10) to compared GFP activity under a non-infected condition. Residual GFP activity was also visible in another control experiment, in which we infected the GFP reporter plants with the oomycete pathogen *Hyaloperonospora arabidopsidis* (new Figure S13).

**Reviewer #1:** Figure S7 legend is very unclear: refers to a scale bar in C, which is apparently a rtQPCR assay, which is never made clear in legend text. Same for panel E.

**Authors’ response:** We thank the reviewer to point this out. We now have clarified the figure legend.

**Reviewer #1:** Figure S7E: there is a difference in BcTUB accumulation between WT and reporter containing Arabidopsis plants. This suggests there is a difference on the pathogenicity of the fugus imparted by the construct, unless different amounts were inoculated.

**Authors’ response:** We have now re-analyzed fungal biomass by measuring the *B. cinerea* genomic DNA content instead of *BcTUB* mRNA levels, which is a more appropriate way to quantify pathogen load in infected plant tissue. This new data indicated no significant difference when infecting *A. thaliana* WT or GFP reporter plants.

**Reviewer #1:** Line 116-117: in depth?

**Authors’ response:** We deleted “in depth” to make the sentence clearer.

**Reviewer #1:** Line 130: Vacuolar to replace Valuolar.

**Authors’ response:** We corrected this mistake.

**Reviewer #2:** Line 136 to 139: This sentence is complicated and difficult to follow. It needs to be divided into simple sentences. “When infecting S. lycopersicum or A. thaliana with B. cinerea bcrdr1 ko mutants, down-regulation of Bc-sRNA target genes, as observed in WT-infected plants infected with the wild-type strain compared to non-inoculated plants, was abolished or less strong (Figure 3C-D, Figure S5A-B).”

**Authors’ response:** We now have split this sentence into two.

**Reviewer #2:** Figure 3B: Please state which Tubulin gene was used as the reference gene, α-TUB or β-TUB? And for future reference, please note that UBQ appears to show more stable expression than α-TUB/β-TUB as a reference gene in B. cinerea. Please see: Ren H, Wu X, Lyu Y, Zhou H, Xie X, Zhang X, Yang H. Selection of reliable reference genes for gene expression studies in Botrytis cinerea. J Microbiol Methods. 2017 Nov;142:71-75. doi: 10.1016/j.mimet.2017.09.006. Epub 2017 Sep 14. PMID: 28917607.

**Authors’ response:** We thank this reviewer for the recommendation. We have now changed from measuring *BcTubA* mRNAs by qRT-PCR to measuring genomic DNA by qPCR using *BcTubA* primers. We now include the *BcTubA* gene ID in our materials and methods section and the Table S1.

**Reviewer #2:** Table S1: The first sheet name appears to be in another language, possibly Chinese. Please change it to English.

**Authors’ response:** We have now corrected this.

**Reviewer #2:** Figure S6 panel 1: It appears that for brdcl1/2, the GFP panel and bright field (BF) panel are from different imaging. Although there is no GFP signal, you should be able to see the same cell pattern in both the GFP and BF images when changing the contrast of the images. Please carefully review all microscopy images.

**Authors’ response:** We thank this reviewer for this comment. We now have reviewed all microscopy images for any mistake.

**Reviewer #3:** Lines 38-39: “The cellular-type RDRs are conserved in several eukaryote kingdoms, including plants, nematodes, and fungi”. If using the term “kingdoms”, nematodes should be listed under animals – if wanting to make the point that RDRs are found in some animals like nematodes, but not in e.g. mammals, this should be reworded.

**Authors’ response:** We now have changed “nematodes” into “animals”.

**Reviewer #3:** Line 88: SPAG- “we” not “were”.

**Authors’ response:** We corrected this mistake.

**Reviewer #3:** Line 89: “Through a genome survey”. What does genome survey mean in this context? Genome survey often refers to genomic structure which I assume the authors are not referring to, thus how were the three RDR-encoding genes identified - by homology to characterised RDRs (e.g. SAD-1/QDE-1), gene annotation searching, protein domain analysis? This specific approach used should be clarified here, and should also be described in Materials and Methods.

**Authors’ response:** We corrected this mistake and now wrote “through a homology search with the *N. crassa* RDR, SAD-1, …” Details of the BLASTp search are now given in the materials and methods section.

**Reviewer #3:** On a related note, I would advise the authors to include greater detail with respect to any in silico analyses (both this and phylogenetic analysis) in the supplementary materials. While gene loci identifiers are provided throughout, a table describing the source of the sequence data used (e.g. NCBI/Phytozome/ensemblPlants) and the date this resource was accessed will greatly improve replicatibility of the study - as would the transcript IDs used in phylogenetic analysis.

**Authors’ response:** We now include the new Table S2, referring to the amino acid sequences used in the phylogeny analysis.

**Reviewer #3:** Lines 98-99: “however, there was no significant up-regulation of any of the BcRDRs measured during infection (Figure S2A).” In fact, there appears to be significant downregulation of RDR1 at 2dpi, and RDR3 from 1dpi onwards- please could the authors comment on/ discuss the implications thereof?

**Authors’ response:** We now repeated gene expression analysis of *BcRDR*s with a second inoculation experiment by qRT-PCR (updated Figure S2). There was no clear transcriptional regulation of *BcRDR1* when comparing growth in axenic culture and tomato infection. We measured elevated mRNA levels of *BcGypsy*s, as a major source of 21-22 nucleotide long Bc-sRNAs (updated Figure S7D). We therefore suggest that up-regulation of *BcGypsy* precursor mRNAs rather than *BcRDR1* led to increased accumulation of Bc-sRNAs.

**Reviewer #3:** Lines 104-105: “lower fungal biomass, as estimated by quantification of B. cinerea Tubulin mRNAs levels”. The authors later state axenic growth is not impaired in the mutants but it would be reassuring to see that BcTubulin expression is also not affected in the knockout mutants relative to wild type strains to provide additional direct evidence for the authors’ conclusion. Indeed, this would be particularly useful to show in relation to Figure S7E which shows reduced BcTubulin expression in mutants even with 10x greater conidia concentrations used for inoculation.

**Authors’ response:** We now re-analyzed fungal biomass by measuring the *B. cinerea* genomic DNA content, a more appropriate method to quantify pathogen load in infected plant tissue.

**Reviewer #3:** Lines 107-108: “Based on these results, we assume that BcRDR1 is a pathogenicity factor in B. cinerea”. Careful of wording here- “assume” implies a judgement without evidence- better to say “we believe”, or “BcRDR1 is likely a pathogenicity factor in B.cinerea”

**Authors’ response:** We have now re-phrased the sentence.

**Reviewer #3:** Line 113: “B. cinerea Bc-sRNA biogenesis”. Redundant- either “B.cinerea sRNA biogenesis” or “Bc-sRNA biogenesis”.

**Authors’ response:** We removed this redundancy.

**Reviewer #3:** Line 130: SPAG- “Vacuolar” not “valuolar”.

**Authors’ response:** We corrected this mistake.

**Reviewer #3:** Line 133: “and the A. thaliana host genes AtMPK1 (AT1G10210), AtMPK2”. All other genes are given full names here, these should be too.

**Authors’ response:** We now give the full name, *Mitogen-activated protein kinase*.

**Reviewer #3:** Line 134: “Peroxiredoxin” not “Peroxredoxin”.

**Authors’ response:** We corrected this mis-spelling.

**Reviewer #3:** Lines 141-142: “S. lycopersicum Sl Proteinase inhibitor (PI)-I and SlPI-II or A. thaliana AtPlant Defensin (PDF)1.2 immunity genes”. Redundancy- can remove the “Sl” and “At”.

**Authors’ response:** We have fixed this redundancy.

**Reviewer #3:** Paragraph 145-176. Is it correct to claim that this is a “novel” reporter system for ckRNAi- to my knowledge this has been done before in the Dunker et al. 2020 paper- I understand that the GUS has been switched out for GFP in this case but does this really add this much novelty, as it is still operating on the same principles. To be accurate this should be rephrased to better reflect the adaptation of this ckRNAi reporter and the Dunker 2020 paper should be cited as the first instance of using this Csy4-based ckRNAi reporter– also, why was the decision made to switch out GUS for GFP? This does not necessarily need to be explained in the main text but would be interesting to know.

**Authors’ response:** We now removed the word “novel”. We also make the link to the predecessor version and cite Dunker *et al*., 2020. The reason to upgrade for a GFP reporter was to perform life imaging of cross-kingdom RNAi in infected plant tissue.

**Reviewer #3:** Lines 174-176 refer to observations that strongest GFP activity was identified at the infection front after inoculation with WT B. cinerea but, while interesting, it is not immediately apparent how this relates to the overarching results that bcrdr1 mutants are compromised in ckRNAi - was this observation not found upon inoculation with the mutant strains?

**Authors’ response:** While this observation was not directly related to the main finding that *bcrdr1* was incapable to activate the GFP reporter, we feel it is worth mentioning the observation the GFP activity was seen at the infection front, and even more interestingly beyond in plant cell layers that were obviously not colonized by *Botrytis*, yet. This might hint towards a local spreading of Bc-sRNAs into plant cells to prepare the ground for future infection scenarios.

**Reviewer #3:** How did the authors discriminate between increased GFP expression in infected material and increased autofluorescence due to higher fungal infection compared to ko lines. Please could the authors provide controls to discriminate between GFP signal and autofluorescence.

**Authors’ response:** We now included a time series of water-treated GFP reporter plants in the update Figure 4 and new Figure S10 and *B. cinerea* WT-infected *A. thaliana* WT plant seedlings to assess infection-related plant auto-fluorescence (Figure S11). Furthermore, we performed inoculation with a 10x higher conidiospore concentration of the *bcrdr1* ko mutant that compensated for the reduced biomass found in our previous inoculation assays. We now also include another control experiment, in which we infected the *Botrytis*-specific GFP reporter plants with the oomycete pathogen *H. arabidopsidis* (new Figure S13). While Trypan Blue staining confirmed colonization by the oomycete, there was no GFP activated at the infection sites. Note that some GFP fluorescence was seen at the veins of the reporter plant, similar to water-treated GFP reporter plants (Figure 4 and Figure S10), but never at the oomycete infection sites.

**Reviewer #3:** Lines 191-193: “Bc-sRNAs STTM plants exhibited reduced lesion sizes induced by B. cinerea, compared to A. thaliana WT or a transgenic A. thaliana line expressing a non-sense RNA-STTM”. I am assuming that the authors mean a “nonsense” STTM- i.e. one with a scrambled sequence that will not lock the sRNAs- this is made a bit unclear with the hyphenation of “nonsense” to “non-sense” which to me implies something related to sense and antisense strands- I think this should be changed to make the meaning clearer (again in line 195 and in figure 5B, 5C, S8).

**Authors’ response:** We have changed this into “nonsense”.

**Reviewer #3:** Lines 210-211: “A. thaliana master STTM plants grew and developed normally and did not show any pleiotropic defect.” Please could the author show the data?

**Authors’ response:** We now included the new Figure S18 that depicts the growth phenotype of all STTM plant lines used in this study.

**Reviewer #3:** Lines 215-216: “Reduced infection was not due to constantly enhanced plant immunity”. The authors’ conclusion is drawn from evidence that Pseudomonas syringae infection is not affected in the master STTM plants. While this is useful evidence, this is assuming fungal, oomycete and bacterial pathogens all elicit the same immune response. Better evidence for this claim would be to show that the expression of plant immunity-associated genes (other than those targeted by the pathogens) in the master STTM plants is not affected upon inoculation with the assayed pathogens.

**Authors’ response:** We now include gene expression data of the immunity-associated genes *AtPR1* and *AtPDF1.2* in non-infected plants shown in the updated Figure S17D. Expression of both genes were not induced in STTM plants without pathogen treatment.

**Reviewer #3:** Lines 217-218: “did not result in fewer reduced bacterial colonies” -> either fewer or reduced, not both.

**Authors’ response:** We corrected this mistake.

**Reviewer #3:** Lines 230-232: The authors mention the bcdcl1dcl2 ko in the ku70 background that showed no evidence for ckRNAi – what point is the authors making here?

**Authors’ response:** We here comprehensively discuss the findings made on BcDCLs in the context of *B. cinerea* pathogenicity, because BcDCLs, like BcRDRs, are relevant for Bc-sRNA biogenesis. We think it is important to include a recent report that claimed controversy of cross-kingdom RNAi in *B. cinerea*. We here would like to guide the reader to all the relevant references that explain the controversy. In this respect, our study on the BcRDR1 delivers more evidence for the existence and importance of cross-kingdom RNAi for *B. cinerea* pathogenicity.

**Reviewer #3:** Lines 268-270: “It will be interesting to investigate whether microbial RDRs play a broader role in diverse plant-microbe interactions and in cross-kingdom RNA communication.” As the targeting of BcDCLs using SIGS has already been mentioned in this paragraph, it might be nice to further extend this conclusion to suggest how this research could be extended to have some practical applications i.e. the identification of a new fungal pathogenicity factor (BcRDR1) that could similarly be targeted using SIGS in disease control.

**Authors’ response:** We now expanded our conclusion by mentioning RNAi-based crop protection strategies.

**Reviewer #3:** Line 325: “using a binocular.” -> “using a binocular microscope.”

**Authors’ response:** We have corrected this mistake.

**Reviewer #3:** Line 378: “by default multiple alignment algorithms.” While the authors have included the software and version number used in this analysis, this software is proprietary and all readers may not have access to it. It would be nice to at least include what these default settings are, even if just as “... algorithms (gap open cost: X; gap extension cost: Y).” to enable replication using alternative software.

**Authors’ response:** We now included this information in our materials and methods section.

**Reviewer #3:** Line 505: “The scale bars in A) and C) represent 500 μm”. Should this be “A) and B)”?

**Authors’ response:** Yes, we have corrected this mistake.

**Reviewer #3:** Figure S1: This figure would benefit from an explanation of the ‘Max’ and ‘Min’ colour spectrum used within (whether in the caption or by adjusting these labels). Does this refer to residue conservation or another measure of alignment/homology?

**Authors’ response:** We now include this information in the Figure S1 legend.

**Reviewer #3:** Figures S6-S7: The brightfield images should be included as they were taken (i.e. before merging with GFP) such that the figures show BF, GFP, merged.

**Authors’ response:** We now include BF images in the Figure S12.

**Figure R1: Phasing analysis at BcGypsy loci.** A) Profile of aligned read lengths for the locus or cDNA, measured in aligned reads per million (referring to genome alignment). Colors correspond to genotype groups (wildtype in black, bcrdr1 ko mutant in orange). B) Fourier transformed alignment-positions of reads, based on the provided phase-size. RPM and color as above. The 0-position is also re-plotted at the final position, shown by a hollow circle. C) Heatmap showing the Fourier transformation, expanded to include files (rows) from -10 to +10 away from the most expressed position. Color is log-2 scaled to position depth. D) Coverage tracks of alignments from WT and bcrdr1 mutant libraries. Sense and antisense alignments are separated into positive (red) and negative (blue) coverage values, respectively. Values are log10 transformed aligned reads per million, relative to the genome alignment. Arrows indicate the left-alignment-position of the most abundant register and position.

References:

**Dunker, F., Trutzenberg, A., Rothenpieler, J.S., Kuhn, S., Prols, R., Schreiber, T., Tissier, A., Kemen, A., Kemen, E., Huckelhoven, R., and Weiberg, A.** (2020). Oomycete small RNAs bind to the plant RNA-induced silencing complex for virulence. Elife **9,** e56096.

**Guo, Q., Qu, X., and Jin, W.** (2015). PhaseTank: genome-wide computational identification of phasiRNAs and their regulatory cascades. Bioinformatics **31,** 284-286.

**Leisen, T., Werner, J., Pattar, P., Safari, N., Ymeri, E., Sommer, F., Schroda, M., Suarez, I., Collado, I.G., Scheuring, D., and Hahn, M.** (2022). Multiple knockout mutants reveal a high redundancy of phytotoxic compounds contributing to necrotrophic pathogenesis of *Botrytis cinerea*. PLoS Pathog **18,** e1010367.

**Porquier, A., Tisserant, C., Salinas, F., Glassl, C., Wange, L., Enard, W., Hauser, A., Hahn, M., and Weiberg, A.** (2021). Retrotransposons as pathogenicity factors of the plant pathogenic fungus *Botrytis cinerea*. Genome Biol **22,** 225.

**Weiberg, A., Wang, M., Lin, F.M., Zhao, H., Zhang, Z., Kaloshian, I., Huang, H.D., and Jin, H.** (2013). Fungal small RNAs suppress plant immunity by hijacking host RNA interference pathways. Science **342,** 118-123.
